# Design, synthesis, and apoptotic antiproliferative activity of novel dihydropyrimidine-5-carbonitrile/1,2,4-oxadiazole hybrids as dual EGFR/VEGFR-2 inhibitors endowed with antioxidant activity

**DOI:** 10.1039/d5ra05685c

**Published:** 2025-10-16

**Authors:** Lamya H. Al-Wahaibi, Amira M. Mohamed, Hesham A. Abou-Zied, Abdullah Yahya Abdullah Alzahrani, Stefan Bräse, Bahaa G. M. Youssif

**Affiliations:** a Department of Chemistry, College of Sciences, Princess Nourah bint Abdulrahman University Saudi Arabia; b Pharmaceutical Organic Chemistry Department, Faculty of Pharmacy, Assiut University Assiut 71526 Egypt bgyoussif2@gmail.com (002)-01098294419; c Medicinal Chemistry Department, Faculty of Pharmacy, Deraya University Minia Egypt; d Department of Chemistry, Faculty of Science, King Khalid University Abha 61413 Saudi Arabia; e Institute of Biological and Chemical Systems, IBCS-FMS, Karlsruhe Institute of Technology 76131 Karlsruhe Germany braese@kit.edu

## Abstract

A new series of dihydropyrimidine-5-carbonitrile/1,2,4-oxadiazole hybrids (10a–l) was developed as dual inhibitors of EGFR and VEGFR-2. The structures of the newly synthesized compounds were confirmed using ^1^H NMR, ^13^C NMR, and elemental analysis. The novel compounds were evaluated for their antioxidant and antiproliferative apoptotic characteristics. Compounds 10e, 10k, and 10l demonstrated the most potent antiproliferative activity and exhibited more efficacy than the reference erlotinib against both Panc-1 (pancreatic) and MCF-7 (breast) cancer cell lines. Compounds 10k and 10l exhibited the highest potency as EGFR and VEGFR-2 inhibitors, with IC_50_ values of 57 nM and 61 nM against EGFR, respectively, and IC_50_ values of 21 nM and 26 nM for VEGFR-2, respectively. Moreover, compounds 10k and 10l demonstrated promising apoptotic activity through the overexpression of caspases-3, 8, and 9, as well as Bax and p53, and the downregulation of the anti-apoptotic protein Bcl-2. Additionally, compounds 10k and 10l exhibited notable antioxidant activity at 10 μM, demonstrating DPPH radical scavenging rates of 72.5% and 69.8%, respectively. An integrated computational study was conducted to validate the dual kinase inhibitory potential of compound 10k and 10i against EGFR and VEGFR-2. Compound 10k and 10i established strong hydrogen bonds with Met769 in EGFR and Glu885 in VEGFR-2, achieving interaction energies of −8.21 and −7.42 kcal mol^−1^, respectively. Molecular dynamics simulations over 100 ns confirmed that the 10k–kinase complexes remained highly stable, showing minimal conformational fluctuations throughout the simulation. Compound 10i also exhibited stable dynamics and favorable interactions; however, 10k consistently maintained stable binding conformations. These results highlight 10k as the most dynamically stable and potent dual EGFR/VEGFR-2 inhibitor in the series. DFT analysis revealed a moderate HOMO–LUMO gap (3.86 eV), chemical hardness (1.93 eV), and a dipole moment of 6.4 debye, which correlates with favorable reactivity and polarity for kinase engagement. ADME profiling highlighted drug-likeness, acceptable bioavailability, and selective CYP inhibition. Altogether, these findings validate 10k as a promising dual EGFR/VEGFR-2 inhibitor with strong structural and pharmacokinetic potential.

## Abstract 2

A series of new dihydropyrimidine/1,2,4-oxadiazole compounds was designed and synthesised. Their structures were validated by IR, NMR, and elemental analysis, and they were evaluated as antiproliferative agents targeting EGFR and VEGFR-2.

## Graphical abstract

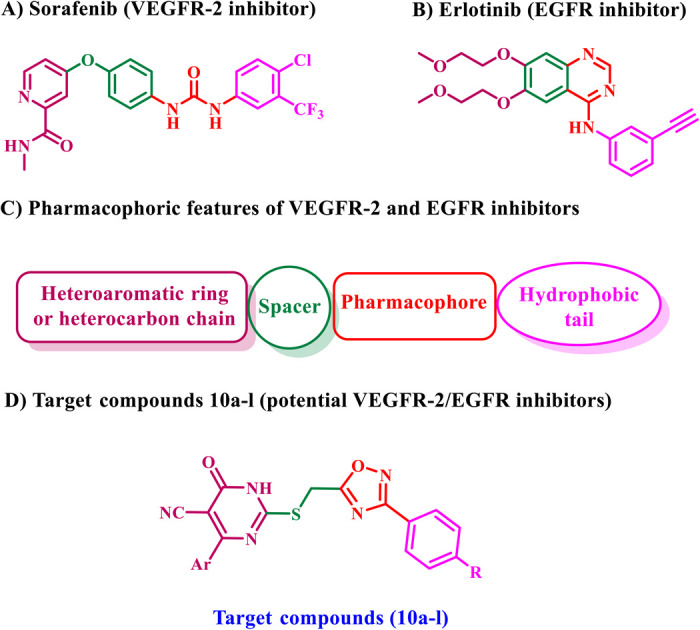

## 1 Introduction

Despite substantial advancements in this domain, cancer treatment remains one of the most significant medical issues. The cancer issue increasingly impacts low- and middle-income countries, as well as impoverished individuals across all nations, illustrating the inherent socio–economic correlation.^1,2^ Cancer is the second leading cause of mortality globally, surpassed only by cardiovascular illnesses. The World Health Organization (WHO) states that cancer is the second foremost cause of mortality globally, responsible for 9.6 million deaths in 2018.^3,4^ The projected increase in the number of affected individuals over the next two decades is around 70%.^5^

Cancer develops when normal cells forfeit their regulatory mechanisms governing proliferation.^6^ Protein kinases (PKs) are essential for regulating physiological activities, including cell proliferation, metabolism, survival, and apoptosis. These enzymes catalyze the transfer of the γ-phosphate group from ATP to specific threonine, serine, or tyrosine hydroxyl groups on target protein substrates implicated in various cellular signaling cascades.^7,8^ Disruption of cell signaling pathways *via* kinase modifications (notably hyper-activation, hyper-production, or mutation) results in several health problems, including cancer.^9^

Targeted chemotherapy has emerged to overcome the resistance and undesirable side effects associated with conventional, non-selective anticancer drugs, utilizing various approaches such as apoptosis induction and angiogenesis suppression.^10^ The earliest approved targeted medicines include the class of protein tyrosine kinase inhibitors (PTKIs). Receptor tyrosine kinases (RTKs) are cell surface receptors that are more commonly affected by oncogenic modifications.^11^ Receptor tyrosine kinases (RTKs) play a crucial role in regulating various cellular processes, including cell cycle progression and apoptosis, in both normal and pathological environments.^12,13^ Epidermal growth factor receptor (EGFR) and vascular endothelial growth factor receptor (VEGFR-2) are classified as receptor tyrosine kinases (RTKs).^14^ EGFR plays a crucial function in regulating numerous biological activities, including cell survival, proliferation, and migration.^15,16^ Conversely, VEGFs are distinguished as one of the most specific and essential signaling pro-angiogenic factors implicated in angiogenesis across many human malignancies.^17^ Numerous clinically licensed anticancer drugs exhibit significant inhibitory effect against EGFR and VEGFR-2, including erlotinib, lapatinib, sorafenib, and sunitinib.^18–20^ Consequently, the inhibition of the EGFR and VEGFR-2 signaling pathways has emerged as a compelling technique for the development of novel antiproliferative agents.

In medicinal chemistry, several heterocyclic scaffolds play a pivotal role in the identification of new pharmaceuticals. Pyrimidines, like all heterocyclic compounds, have garnered significant attention due to their fundamental role as components of nucleic acids and their involvement in all living cells. Owing to its significance, it possesses many biological features and applications in pharmacological research. Diverse substituents of pyrimidine-5-carbonitrile exhibit varying therapeutic activity, including anticancer properties. Furthermore, a literature review indicated that the thiopyrimidine-5-carbonitrile ring system has played a significant role in the design and synthesis of innovative chemotherapeutic drugs with notable anticancer properties.^21,22^

**Fig. 1:**
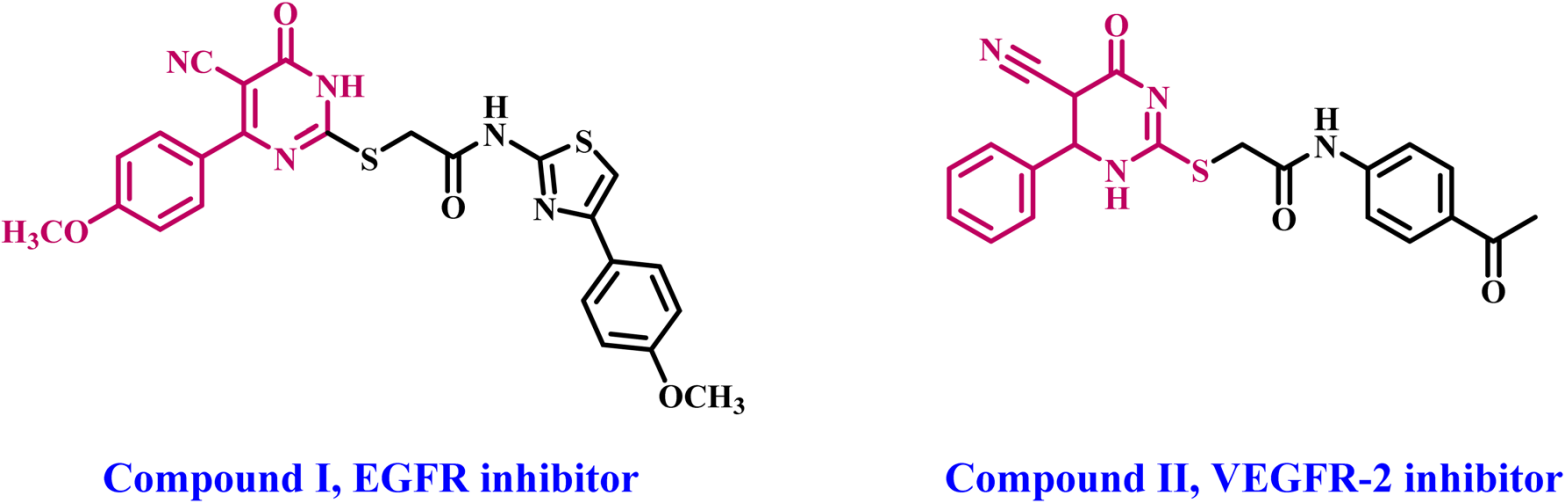
Structures of some reported thiopyrimidine-5-carbonitriles I and II as RTKs.

In 2021,^23^ we present the design, synthesis, and antiproliferative activity of a novel series of thiopyrimidine-5-carbonitrile derivatives hybridized with the 1,3-thiazole moiety. The newly synthesized compounds were evaluated for their antiproliferative effects against four distinct cancer cell lines. Compound I (Fig. 1) was identified as the most potent antiproliferative derivative. The *in vitro* EGFR inhibitory assay results indicated that compound I was also the most efficient EGFR inhibitor, with an IC_50_ value of 0.19 ± 0.20 μM, in contrast to the reference erlotinib, which had an IC_50_ of 0.08 μM. The docking analysis results demonstrate the crucial influence of the methoxy substituent on pyrimidinone-phenyl and cyano nitrogen in binding to the essential amino acids analogous to those associated with the reference compound erlotinib.

In another study,^17^ A number of novel 1,6-dihydropyrimidin-2-thiol compounds as potential VEGFR-2 inhibitors have been designed and synthesized. The National Cancer Institute has selected some of the newly synthesized compounds for *in vitro* anticancer screening. Compound II (Fig. 1) had exceptional anticancer efficacy against the majority of the cell lines tested, including total cell death in leukemia, non-small cell lung cancer, and breast cancer cell lines. *In vitro* five-dose assays demonstrated that compound II had significant activity against the majority of the evaluated cell lines, with GI_50_ values ranging from 19 to 100 μM. Compound II had the highest potency as a VEGFR-2 kinase inhibitor, with an IC_50_ value of 198.7 nM, in contrast to sorafenib, which has an IC_50_ of 0.17 nM. The docking study results demonstrated an acceptable fit of the novel compounds to the active region of VEGFR-2.

Conversely, literature surveys indicate that 1,2,4-oxadiazoles possess statistical relevance in bioorganic and pharmaceutical chemistry. They have been recognized for their varied pharmacological properties.^24–26^ The 1,2,4-oxadiazole has bioisosteric equivalency to ester and amide groups. In the presence of unstable conditions (*e.g.*, hydrolysis), 1,2,4-oxadiazole serves as a very efficacious alternative.^27^ The significant biological effect of 1,2,4-oxadiazole derivatives on cancer cells is due to multiple mechanisms of action.

**Fig. 2:**
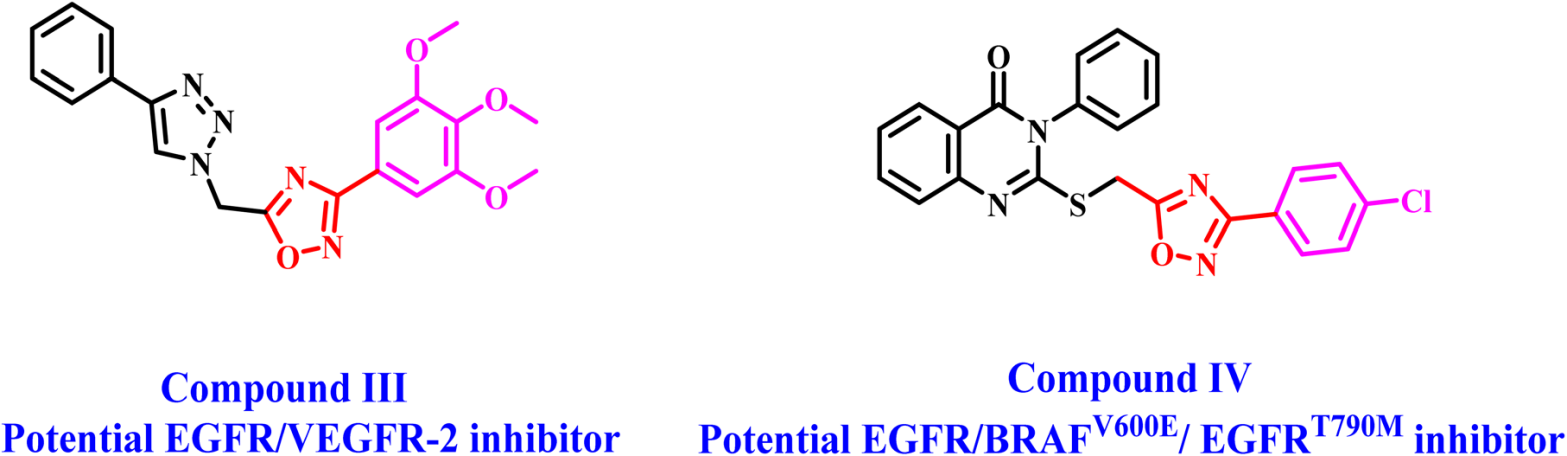
Structures of some 1,2,4-oxadiazole-based derivatives III and IV as PKs.

In a recent paper,^28^ we introduced a novel class of 1,2,4-oxadiazole/1,2,3-triazole hybrids developed as dual inhibitors of EGFR and VEGFR-2. The novel compounds were assessed for their antiproliferative properties, using erlotinib as the reference medication. The results indicated that the majority of the evaluated drugs had substantial antiproliferative activity, with GI_50_ values between 28 and 104 nM, in comparison to erlotinib (GI_50_ = 33 nM). Compound III (Fig. 2) was identified as the most powerful inhibitor of EGFR and VEGFR-2, with IC_50_ values of 76 nM for EGFR and 2.4 nM for VEGFR-2. Compound III induces apoptosis by activating caspase-3, 8, and Bax while down-regulating the anti-apoptotic protein Bcl-2.

In another study from our lab,^29^ we reported the synthesis and antiproliferative activity of some new 1,2,4-oxadiazole/quinazoline hybrids as multi-targeted inhibitors. The results indicated that most of the evaluated compounds exhibited significant antiproliferative effects. *In vitro* assays demonstrated that compound IV is a potent antiproliferative agent, potentially functioning as a dual inhibitor of EGFR and BRAF^V600E^. Compound IV displayed IC_50_ values of 57 nM and 48 nM against EGFR and BRAF^V600E^, respectively. Furthermore, compound IV showed considerable efficacy against mutant EGFR (EGFR^T790M^). Cell cycle analysis and apoptosis detection revealed that compound IV induces cell cycle arrest at the G2/M transition.

### 1.1. Rational design

**Fig. 3:**
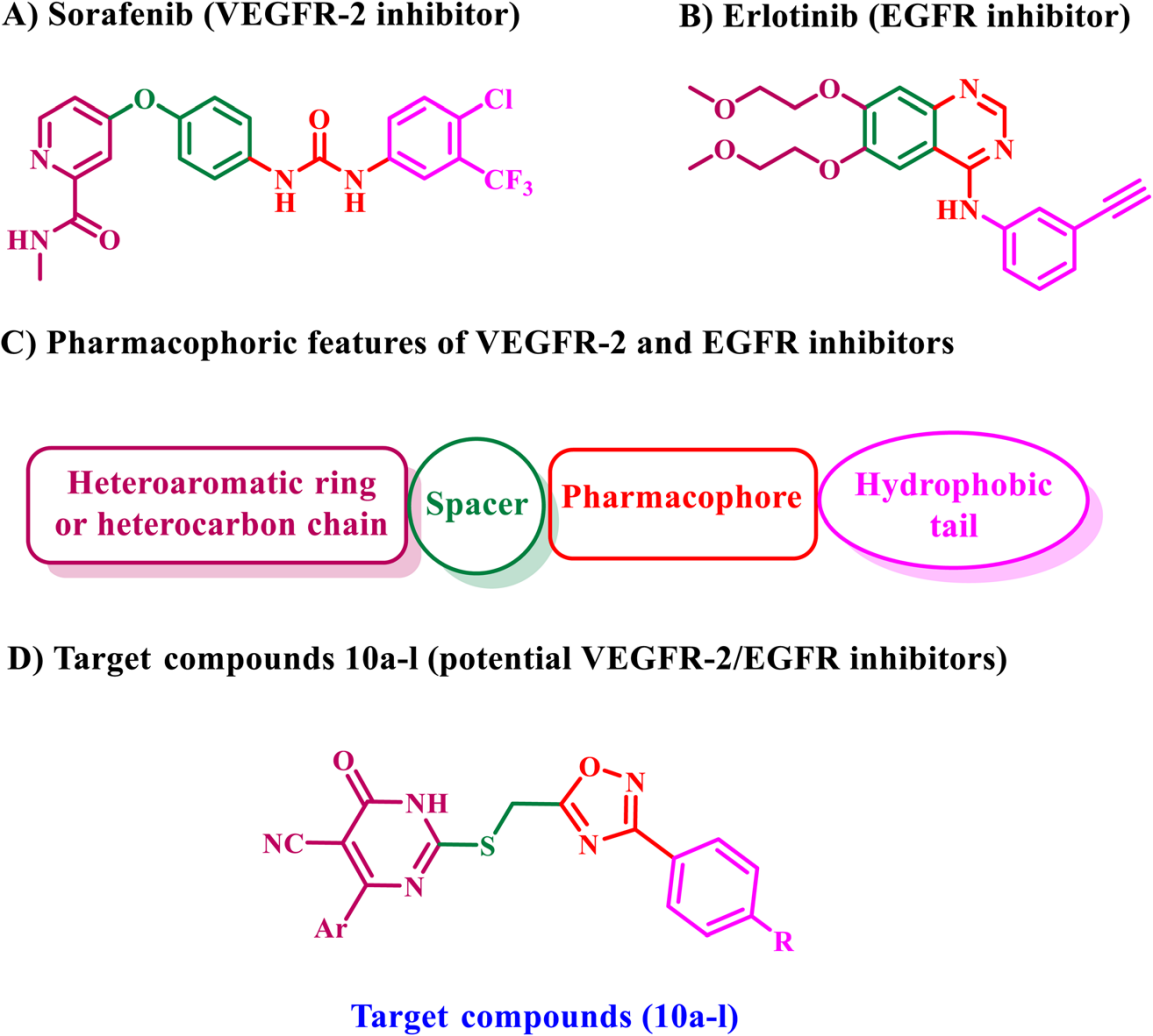
Rational design for compounds 10a–l as dual EGFR/VEGFR-2 inhibitors. (A) Sorafenib; (B) erlotinib; (C) pharmacophoric features of EGFR and VEGFR-2; (D) new targets.

Recent studies have shown that the structure of FDA-approved VEGFR-2 inhibitors has four common characteristics: (a) a heterocyclic aromatic ring that resides in the receptor's hinge region, (b) a spacer that interacts with the gatekeeper region, (c) a hydrogen bonding moiety that forms critical hydrogen bonds with the DFG amino acids, and (d) a hydrophobic tail that occupies the receptor's allosteric location,^30,31^Fig. 3A and C. On the other hand, the pharmacophoric attributes of FDA-approved EGFR inhibitors include a benzo-heterocyclic ring located within the adenine binding pocket, a hydrogen bond donor or acceptor, a hydrophobic moiety occupying hydrophobic region I, and a hetero carbon chain (hydrophobic tail) interacting with hydrophobic region II (Fig. 3B and C).

As a result, and as part of our ongoing attempts to develop a dual EGFR/VEGFR-2 inhibitor,^29,32–35^ we disclose the synthesis of a new series of dihydropyrimidine-5-carbonitriles coupled with a 1,2,4-oxadiazole moiety, which may serve as potential antiproliferative agents. Fig. 3D shows that the new compounds 10a–l have the necessary pharmacophoric moieties to inhibit both EGFR and VEGFR-2. The newly synthesized compounds were confirmed using ^1^H NMR, ^13^C NMR, and elemental microanalysis. The *in vitro* antiproliferative efficacy of 10a–l was assessed against a panel of four cancer cell lines. The most effective compounds were chosen and subsequently assessed as inhibitors of EGFR and VEGFR-2. Additionally, some compounds were examined for their ability to induce apoptosis by assessing the levels of apoptotic markers, such as caspase-3, -8, and -9, as well as Bax and p53, and the anti-apoptotic protein Bcl-2. Finally, molecular docking and dynamic simulation over 100 ns were performed to investigate the binding interactions and stability of the new compounds within the binding sites of both EGFR and VEGFR-2 proteins.

## 2 Experimental

### 2.1. Chemistry

General details: See Appendix A (SI File) 2-mercapto-6-oxo-4-phenyl-1,6-dihydropyrimidine-5-carbonitriles 4a–d (ref. 23) and 3-aryl-5-(chloromethyl)-1,2,4-oxadiazoles 9a–c,^29,36^ were prepared according to literature methods.

#### 2.1.1. General procedures for the synthesis of new compounds (10a–l)

To a stirred solution of compounds 4a–d (0.60 mmol, 1.0 eq.) in DMF (5 mL), anhydrous K_2_CO_3_ (0.72 mmol, 1.2 eq., 0.10 g) was added and stirred for 1 hour at ambient temperature. Subsequently, 3-aryl-5-(chloromethyl)-1,2,4-oxadiazoles 9a–c (0.60 mmol, 1.0 eq.) and KI (0.90 mmol, 1.5 eq., 0.15 g) were included into the reaction mixture, which was stirred for 24 hours. Upon completion of the reaction (verified by TLC utilizing hexane : ethyl acetate 1 : 2), the reaction mixture was added to crushed ice while stirring. The precipitate was filtered, washed many times with water, dried at 60 °C, and crystallized from ethanol to yield pure compounds 10a–l.

##### 2.1.1.1. 2-(((3-Phenyl-1,2,4-oxadiazol-5-yl)methyl)thio)-6-oxo-4-phenyl-1,6-dihydropyrimidine-5-carbonitrile (10a)

Yield: 0.16 g (70%), white solid, m. p: 151–153 °C. ^1^H NMR (400 MHz, *δ* ppm DMSO-*d*_6_): 8.02 (d, *J* = 4.0 Hz, 2H, Ar–H), 7.74 (d, *J* = 5.2 Hz, 2H, Ar–H), 7.59 (s, 4H, Ar–H), 7.43 (d, *J* = 7.2 Hz, 2H, Ar–H), 4.68 (s, 2H, S–CH_2_); ^13^C NMR (100 MHz, *δ* ppm DMSO-*d*_6_): 178.83, 170.72, 170.18, 168.26, 167.55, 137.79, 132.05, 130.34, 129.74, 128.58, 128.52, 127.47, 126.62, 120.18, 90.07, 25.49; anal. calc. (%) for C_20_H_13_N_5_O_2_S: C, 62.01; H, 3.38; N, 18.08; S, 8.28. Found: C, 62.09; H, 3.42; N, 18.14.

##### 2.1.1.2. 2-(((3-(4-Chlorophenyl)-1,2,4-oxadiazol-5-yl)methyl)thio)-6-oxo-4-phenyl-1,6-dihydropyrimidine-5-carbonitrile (10b)

Yield: 0.18 g (73%), white solid, m. p: 156–158 °C. ^1^H NMR (400 MHz, *δ* ppm DMSO-*d*_6_): 8.02 (d, *J* = 8.6 Hz, 2H, Ar–H *p*-Cl C_6_H_4_), 7.72 (dd, *J* = 8.0, 1.5 Hz, 2H, Ar–H), 7.64 (d, *J* = 8.6 Hz, 2H, Ar–H *p*-Cl C_6_H_4_), 7.46–7.39 (m, 3H, Ar–H), 4.67 (s, 2H, S–CH_2_); ^13^C NMR (100 MHz, *δ* ppm DMSO-*d*_6_): 179.15, 170.58, 170.10, 167.53, 167.48, 137.82, 136.75, 130.31, 129.92, 129.29, 128.56, 128.51, 125.49, 120.21, 90.08, 25.49; anal. calc. (%) for C_20_H_12_ClN_5_O_2_S: C, 56.94; H, 2.87; N, 16.60; S, 7.60. Found: C, 56.88; H, 2.94; N, 16.64.

##### 2.1.1.3. 2-(((3-(4-Methoxyphenyl)-1,2,4-oxadiazol-5-yl)methyl)thio)-6-oxo-4-phenyl-1,6-dihydropyrimidine-5-carbonitrile (10c)

Yield: 0.17 g (68%), white solid, m. p: 161–163 °C. ^1^H NMR (400 MHz, *δ* ppm DMSO-*d*_6_): 8.02 (d, *J* = 4.0 Hz, 2H, Ar–H), 7.77 (d, *J* = 8.0 Hz, 2H, Ar–H, *p*-OCH_3_ C_6_H_4_), 7.62–7.54 (m, 3H, Ar–H), 6.95 (d, *J* = 8.0 Hz, 2H, Ar–H, *p*-OCH_3_ C_6_H_4_), 4.66 (s, 2H, S–CH_2_), 3.80 (s, 3H, O–CH_3_); ^13^C NMR (100 MHz, *δ* ppm DMSO-*d*_6_): 178.95, 170.79, 169.91, 168.22, 166.70, 161.03, 132.09, 130.25, 130.08, 129.79, 127.45, 126.60, 120.64, 113.80, 89.23, 55.77, 25.45; anal. calc. (%) for C_21_H_15_N_5_O_3_S: C, 60.42; H, 3.62; N, 16.78; S, 7.68. Found: C, 60.48; H, 3.50; N, 16.82.

##### 2.1.1.4. 2-(((3-Phenyl-1,2,4-oxadiazol-5-yl)methyl)thio)-6-oxo-4-(pyridin-4-yl)-1,6-dihydropyrimidine-5-carbonitrile (10d)

Yield: 0.16 g (69%), orange solid, m. p: 152–154 °C. ^1^H NMR (400 MHz, *δ* ppm DMSO-*d*_6_): 8.66 (d, *J* = 6.0 Hz, 2H, Ar–H), 8.01 (dd, *J* = 7.0, 1.5 Hz, 2H, Ar–H), 7.69 (d, *J* = 6.0 Hz, 2H, Ar–H), 7.60–7.55 (m, 3H, Ar–H), 4.67 (s, 2H, S–CH_2_); ^13^C NMR (100 MHz, *δ* ppm DMSO-*d*_6_): 178.74, 170.67, 170.03, 168.25, 165.26, 150.23, 144.91, 132.07, 129.75, 127.46, 126.57, 122.79, 119.49, 90.63, 25.53; anal. calc. (%) for C_19_H_12_N_6_O_2_S: C, 58.76; H, 3.11; N, 21.64; S, 8.25. Found: C, 58.82; H, 3.09; N, 21.70.

##### 2.1.1.5. 2-(((3-(4-Chlorophenyl)-1,2,4-oxadiazol-5-yl)methyl)thio)-6-oxo-4-(pyridin-4-yl)-1,6-dihydropyrimidine-5-carbonitrile (10e)

Yield: 0.18 g (72%), orange solid, m. p: 157–159 °C. ^1^H NMR (400 MHz, *δ* ppm DMSO-*d*_6_): 8.66 (d, *J* = 4.0 Hz, 2H, Ar–H), 8.01 (d, *J* = 8.0 Hz, 2H, Ar–H *p*-Cl C_6_H_4_), 7.67 (d, *J* = 4.0 Hz, 2H, Ar–H), 7.65 (d, *J* = 8.0 Hz, 2H, Ar–H *p*-Cl C_6_H_4_), 4.67 (s, 2H, S–CH_2_); ^13^C NMR (100 MHz, *δ* ppm DMSO-*d*_6_): 179.04, 170.60, 169.99, 167.47, 165.23, 150.25, 144.89, 136.76, 129.94, 129.28, 125.45, 122.76, 119.48, 90.62, 25.54; anal. calc. (%) for C_19_H_11_ClN_6_O_2_S: C, 53.97; H, 2.62; N, 19.88; S, 7.58. Found: C, 54.06; H, 2.70; N, 19.79.

##### 2.1.1.6. 2-(((3-(4-Methoxyphenyl)-1,2,4-oxadiazol-5-yl)methyl)thio)-6-oxo-4-(pyridin-4-yl)-1,6-dihydropyrimidine-5-carbonitrile (10f)

Yield: 0.17 g (68%), orange solid, m. p: 163–165 °C. ^1^H NMR (400 MHz, *δ* ppm DMSO-*d*_6_): 8.69 (d, *J* = 6.0 Hz, 2H, Ar–H), 7.71 (d, *J* = 8.6 Hz, 2H. Ar–H *p*-OCH_3_ C_6_H_4_), 7.68 (d, *J* = 6.0 Hz, 2H, Ar–H), 7.65 (d, *J* = 8.6 Hz, 2H. Ar–H *p*-OCH_3_ C_6_H_4_), 4.67 (s, 2H, S–CH_2_), 3.80 (s, 3H, O–CH_3_); ^13^C NMR (100 MHz, *δ* ppm DMSO-*d*_6_): 178.71, 170.64, 170.03, 168.25, 165.26, 150.23, 144.89, 132.07, 129.75, 127.42, 122.72, 119.44, 113.83, 90.63, 55.70, 25.53; anal. calc. (%) for C_20_H_14_N_6_O_3_S: C, 57.41; H, 3.37; N, 20.09; S, 7.66. Found: C, 57.49; H, 3.29; N, 20.16.

##### 2.1.1.7. 2-(((3-Phenyl-1,2,4-oxadiazol-5-yl)methyl)thio)-4-(4-chlorophenyl)-6-oxo-1,6-dihydropyrimidine-5-carbonitrile (10g)

Yield: 0.19 g (75%), white solid, m. p: 156–158 °C. ^1^H NMR (400 MHz, *δ* ppm DMSO-*d*_6_): 7.99 (d, *J* = 7.2 Hz, 2H, Ar–H), 7.77 (d, *J* = 8.4 Hz, 2H, Ar–H *p*-Cl C_6_H_4_), 7.61–7.56 (m, 3H, Ar–H), 7.48 (d, *J* = 8.4 Hz, 2H, Ar–H *p*-Cl C_6_H_4_), 4.67 (s, 2H, S–CH_2_); ^13^C NMR (100 MHz, *δ* ppm DMSO-*d*_6_): 178.75, 170.74, 170.43, 168.24, 166.34, 136.39, 135.21, 132.08, 130.40, 129.74, 128.66, 127.44, 126.51, 119.87, 90.02, 25.54; anal. calc. (%) for C_20_H_12_ClN_5_O_2_S: C, 56.94; H, 2.87; N, 16.60; S, 7.60. Found: C, 57.03; H, 2.82; N, 16.68.

##### 2.1.1.8. 2-(((3-(4-Chlorophenyl)-1,2,4-oxadiazol-5-yl)methyl)thio)-4-(4-chloro phenyl)-6-oxo-1,6-dihydropyrimidine-5-carbonitrile (10h)

Yield: 0.21 g (78%), white solid, m. p: 162–164 °C. ^1^H NMR (400 MHz, *δ* ppm DMSO-*d*_6_): 8.00 (d, *J* = 8.5 Hz, 2H, Ar–H *p*-Cl C_6_H_4_), 7.76 (d, *J* = 8.5 Hz, 2H, Ar–H *p*-Cl C_6_H_4_), 7.64 (d, *J* = 8.5 Hz, 2H, Ar–H *p*-Cl C_6_H_4_), 7.49 (d, *J* = 8.5 Hz, 2H, Ar–H *p*-Cl C_6_H_4_), 4.66 (s, 2H, S–CH_2_); ^13^C NMR (100 MHz, *δ* ppm DMSO-*d*_6_): 179.15, 170.58, 170.10, 167.53, 167.48, 137.82, 136.75, 130.31, 129.92, 129.29, 128.56, 128.51, 125.49, 120.21, 90.08, 25.49; anal. calc. (%) for C_20_H_11_Cl_2_N_5_O_2_S: C, 52.65; H, 2.43; N, 15.35; S, 7.03. Found: C, 52.69; H, 2.40; N, 15.31.

##### 2.1.1.9. 2-(((3-(4-Methoxyphenyl)-1,2,4-oxadiazol-5-yl)methyl)thio)-4-(4-chloro phenyl)-6-oxo-1,6-dihydropyrimidine-5-carbonitrile (10i)

Yield: 0.20 g (74%), white solid, m. p: 163–165 °C. ^1^H NMR (400 MHz, *δ* ppm DMSO-*d*_6_): 8.02 (d, *J* = 8.6 Hz, 2H, Ar–H *p*-Cl C_6_H_4_), 7.76 (d, *J* = 8.8 Hz, 2H, Ar–H *p*-OCH_3_ C_6_H_4_), 7.65 (d, *J* = 8.6 Hz, 2H, Ar–H *p*-Cl C_6_H_4_), 6.96 (d, *J* = 8.8 Hz, 2H. Ar–H *p*-OCH_3_ C_6_H_4_), 4.66 (s, 2H, S–CH_2_), 3.80 (s, 3H, O–CH_3_); ^13^C NMR (100 MHz, *δ* ppm DMSO-*d*_6_): 179.17, 170.84, 169.86, 167.44, 166.76, 161.09, 136.73, 130.14, 130.08, 129.96, 129.27, 125.48, 120.56, 113.81, 89.20, 55.76, 25.47; anal. calc. (%) for C_21_H_14_ClN_5_O_3_S: C, 55.82; H, 3.12; N, 15.50; S, 7.09. Found: C, 55.86; H, 3.19; N, 15.53.

##### 2.1.1.10. 2-(((3-Phenyl-1,2,4-oxadiazol-5-yl)methyl)thio)-4-(4-methoxyphenyl)-6-oxo-1,6-dihydropyrimidine-5-carbonitrile (10j)

Yield: 0.18 g (72%), yellow solid, m. p: 162–164 °C. ^1^H NMR (400 MHz, *δ* ppm DMSO-*d*_6_): 8.01 (d, *J* = 4.6 Hz, 2H, Ar–H), 7.76 (d, *J* = 8.2 Hz, 2H, Ar–H *p*-OCH_3_ C_6_H_4_), 7.62–7.54 (m, 3H, Ar–H), 6.95 (d, *J* = 8.2 Hz, 2H, Ar–H *p*-OCH_3_ C_6_H_4_), 4.66 (s, 2H, S–CH_2_), 3.80 (s, 3H, O–CH_3_); ^13^C NMR (100 MHz, *δ* ppm DMSO-*d*_6_): 178.90, 170.78, 169.93, 168.24, 166.73, 161.07, 132.03, 130.20, 130.05, 129.73, 127.48, 126.65, 120.60, 113.85, 89.20, 55.71, 25.50; anal. calc. (%) for C_21_H_15_N_5_O_3_S: C, 60.42; H, 3.62; N, 16.78; S, 7.68. Found: C, 60.40; H, 3.69; N, 16.84.

##### 2.1.1.11. 2-(((3-(4-Chlorophenyl)-1,2,4-oxadiazol-5-yl)methyl)thio)-4-(4-methoxy phenyl)-6-oxo-1,6-dihydropyrimidine-5-carbonitrile (10k)

Yield: 0.19 g (71%), yellow solid, m. p: 167–169 °C. ^1^H NMR (400 MHz, *δ* ppm DMSO-*d*_6_): 8.03 (d, *J* = 8.6 Hz, 2H, Ar–H *p*-Cl C_6_H_4_), 7.76 (d, *J* = 8.8 Hz, 2H, Ar–H *p*-OCH_3_ C_6_H_4_), 7.64 (d, *J* = 8.6 Hz, 2H, Ar–H *p*-Cl C_6_H_4_), 6.96 (d, *J* = 8.8 Hz, 2H. Ar–H *p*-OCH_3_ C_6_H_4_), 4.66 (s, 2H, S–CH_2_), 3.80 (s, 3H, O–CH_3_); ^13^C NMR (100 MHz, *δ* ppm DMSO-*d*_6_): 179.19, 170.81, 169.89, 167.46, 166.73, 161.07, 136.75, 130.18, 130.03, 129.91, 129.30, 125.50, 120.59, 113.85, 89.19, 55.71, 25.50; anal. calc. (%) for C_21_H_14_ClN_5_O_3_S: C, 55.82; H, 3.12; N, 15.50; S, 7.09. Found: C, 55.78; H, 3.10; N, 15.58.

##### 2.1.1.12. 2-(((3-(4-Methoxyphenyl)-1,2,4-oxadiazol-5-yl)methyl)thio)-4-(4-methoxy phenyl)-6-oxo-1,6-dihydropyrimidine-5-carbonitrile (10l)

Yield: 0.18 g (67%), yellow solid, m. p: 172–174 °C. ^1^H NMR (400 MHz, *δ* ppm DMSO-*d*_6_): 7.77 (d, *J* = 8.4 Hz, 2H, Ar–H *p*-OCH_3_ C_6_H_4_), 7.71 (d, *J* = 8.4 Hz, 2H, Ar–H *p*-OCH_3_ C_6_H_4_), 6.98 (d, *J* = 8.4 Hz, 2H, Ar–H *p*-OCH_3_ C_6_H_4_), 6.92 (d, *J* = 8.4 Hz, 2H, Ar–H *p*-OCH_3_ C_6_H_4_), 4.66 (s, 2H, S–CH_2_), 3.79 (s, 3H, O–CH_3_), 3.77 (s, 3H, O–CH_3_); ^13^C NMR (100 MHz, *δ* ppm DMSO-*d*_6_): 178.90, 170.78, 169.93, 168.24, 166.73, 161.07, 161.01, 132.03, 130.20, 129.73, 127.48, 120.60, 114.00, 113.85, 89.20, 55.71, 55.68, 25.50; anal. calc. (%) for C_22_H_17_N_5_O_4_S: C, 59.05; H, 3.83; N, 15.65; S, 7.16. Found: C, 59.14; H, 3.80; N, 15.70.

### 2.2. Biology

#### 2.2.1. Cell viability assay

The impact of novel compounds 10a–l on the viability of a normal human cell line, specifically human mammary gland epithelial cells (MCF-10A), was assessed to ascertain the safety of the newly synthesized compounds. The MTT test was employed to assess the cell viability of the novel compounds after a four-day incubation with MCF-10A cells.^29,37^ Refer to Appendix A for more experimental details.

#### 2.2.2. Antiproliferative assay

The MTT assay was used to determine the antiproliferative effects of compounds 10a–l on four human cancer cell lines using erlotinib as the reference drug. The dose–response experiments established the IC_50_ values for the new compounds. The given findings were obtained from at least two distinct studies, each comprising three repeats per concentration. Appendix A (SI File) provides experimental details.^38,39^

#### 2.2.3. EGFR inhibitory assay

Using the EGFR-TK test, the most potent antiproliferative derivatives 10e, 10i, 10h, 10k, and 10l were evaluated for their capacity to inhibit EGFR. erlotinib was the reference substance.^33,40^ See Appendix A for more details.

#### 2.2.4. BRAF^V600E^ inhibitory assay

Utilizing sorafenib as a reference, the most effective antiproliferative derivatives (10e, 10i, 10h, 10k, and 10l) were evaluated for their ability to inhibit VEGFR-2.^28,35^ Refer to Appendix A for experimental details.

#### 2.2.5. Apoptotic markers assay

Compounds10k and 10l were tested as caspases-3, 8, 9, Bax and p53 activators and as down-regulators of the anti-apoptotic protein Bcl2 against the MCF-7 breast cancer cell line.^41^ Appendix A gives more details.

#### 2.2.6. Antioxidant activity

The scavenging of stable free radicals by 2,2-diphenyl-1-picrylhydrazyl (DPPH) was used to investigate the potential antioxidant properties of compounds 10k and 10l, using Trolox as a control.^42^ See Appendix A for more experimental details.

### 2.3. Computational investigations

Molecular docking simulations for EGFR (PDB ID: 1M17) and VEGFR-2 (PDB ID: 3WZE) were validated *via* a redocking test, wherein the structures of the test proteins were held in a fixed state while the co-crystallized ligands (erlotinib for EGFR and Sorafenib for VEGFR-2) were redocked into their corresponding crystal-binding pockets. See Appendix A for SI.

## 3 Results and discussion

### 3.1. Chemistry

**Scheme 1:**
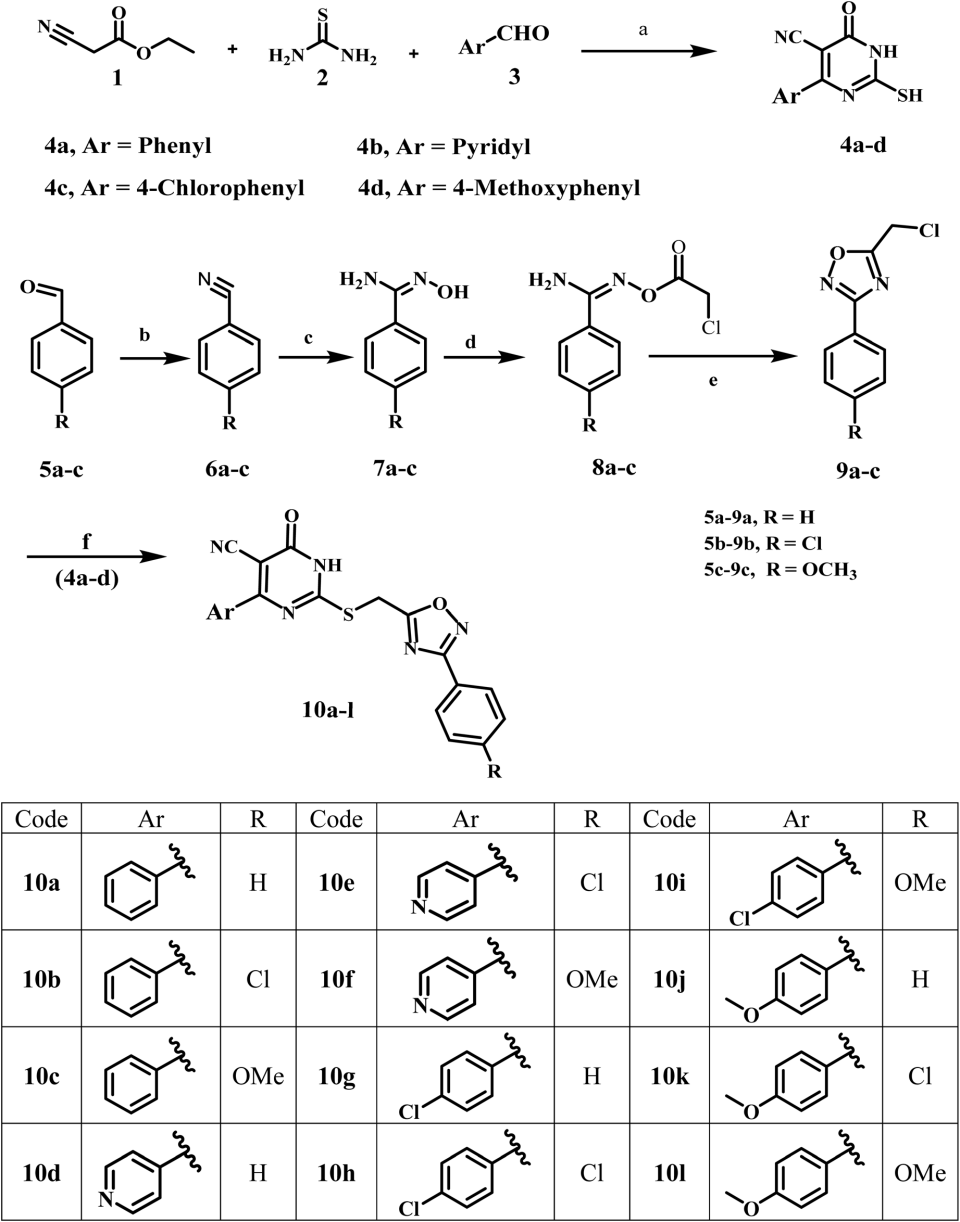
Synthesis of compounds 10a–l. Reagents and conditions: (a) dry anhydrous K_2_CO_3_, ethanol, reflux 18–24 h (80–89%); (b) ammonia (28%), I_2_, THF, stirring 2–3 h (76–80%); (c) NH_2_OH·HCl, Na_2_CO_3_, THF, reflux 12–18 h (50–60%); (d) chloroacetyl chloride, K_2_CO_3_, dry acetone, stirring 24 h (71–77%); (e) toluene, reflux 10–12 h (51–54%); (f) K_2_CO_3_, KI, DMF, stirring 24 h (78–67%).

Scheme 1 summarizes the synthetic pathways of the new target compounds 10a–l. Compounds 4a–d were synthesized by heating a solution of ethyl cyanoacetate (1), thiourea (2), and the corresponding aldehyde (3a–d) in ethanol. The reaction mixture was then heated under reflux for 18–24 h and monitored using TLC. The creamy precipitate was dissolved in hot water and acidified with glacial acetic acid, yielding the desired compounds 4a–d, which were recrystallized from ethanol.^23^ On the other hand, compounds 7a–c, amidoxime derivatives, were synthesized in 50–60% yields over two steps. The first step involved reacting the corresponding aldehydes 5a–c with 28% aqueous ammonia and iodine in THF for 2–3 h to yield the corresponding aryl nitrile derivatives 6a–c in 76–80%.^36^ The second step was a 12 to 18 hours methanol reflux of compounds 6a–c with hydroxylamine chloride and sodium carbonate. Compounds 7a–c were reacted with chloroacetyl chloride in dry acetone to yield benzimidamides (8a–c), which were cyclized by refluxing in toluene to the corresponding 3-aryl-5-(chloromethyl)-1,2,4-oxadiazole derivatives 9a–c as a yellow oil. Compounds 9a–c were purified using column chromatography with hexane : ethyl acetate (9 : 1) as an eluent.^29^

The novel compounds 10a–l were synthesized *via* the reaction of 2-mercapto-6-oxo-4-phenyl-1,6-dihydropyrimidine-5-carbonitriles 4a–d with 3-aryl-5-(chloromethyl)-1,2,4-oxadiazoles 9a–c in DMF, utilizing K_2_CO_3_ and KI as catalysts. The reaction mixture was stirred for 24 hours. Following the reaction's completion, the reaction mixture was added to crushed ice while being stirred. The precipitate obtained was recrystallized from ethanol to produce pure compounds 10a–l.

The structures of novel 10a–l were validated using ^1^NMR, ^13^C NMR, and elemental microanalysis along with FTIR for a representative example. The FTIR spectrum of 10c confirmed the presence of characteristic peaks of the NH group at *ῡ* 3451 cm^−1^, cyano group at *ῡ* 2201 cm^−1^ and carbonyl group at *ῡ* 1589 cm^−1^ as illustrated in Fig. S30 (SI File). The ^1^H NMR spectrum of compound 10l (Ar = 4-OMe-Ph, R = OMe) reveals a distinctive signal of the methylthio spacer as two protons from the CH_2_ group as a singlet signal at *δ* 4.66 ppm. Two singlet signals each of three protons at *δ* 3.79 and *δ* 3.77 ppm correspond to two methoxy groups. In addition, the spectrum revealed two doublets of doublet signals, corresponding to two *para*-disubstituted benzene rings. The ^13^C NMR spectrum confirmed the 10l structure, revealing a singlet signal at *δ* 166.73 ppm for the amidic carbonyl group, a singlet signal at *δ* 120.60 ppm for the nitrile group, two singlet signals at *δ* 55.71 and 55.68 ppm for two methoxy groups, and a singlet signal at *δ* 25.50 ppm for the methylene linker.

**Fig. 4:**
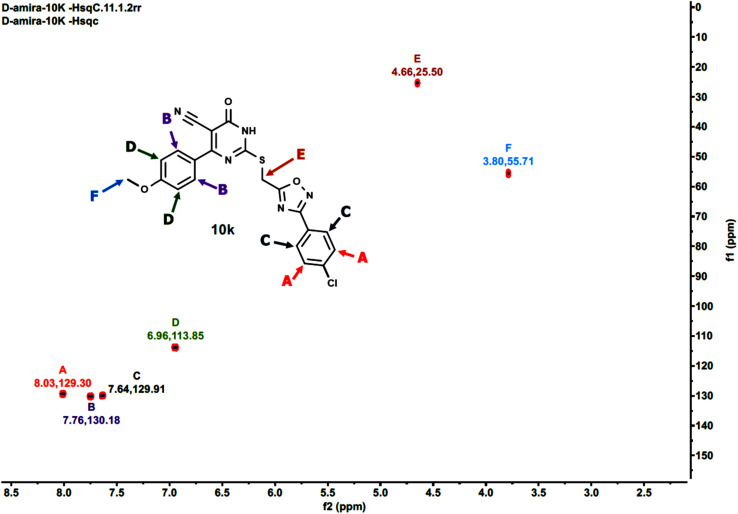
HSQC-2D NMR of 10k.

**Fig. 5:**
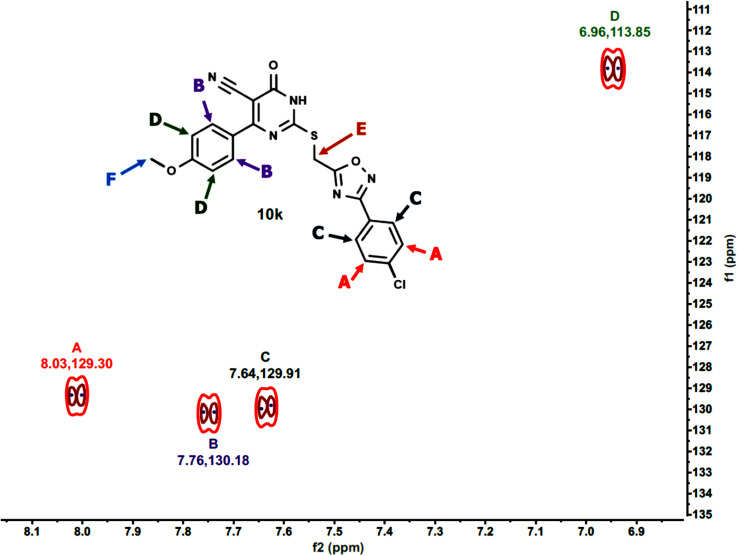
Expanded HSQC-2D NMR of 10k.

**Fig. 6:**
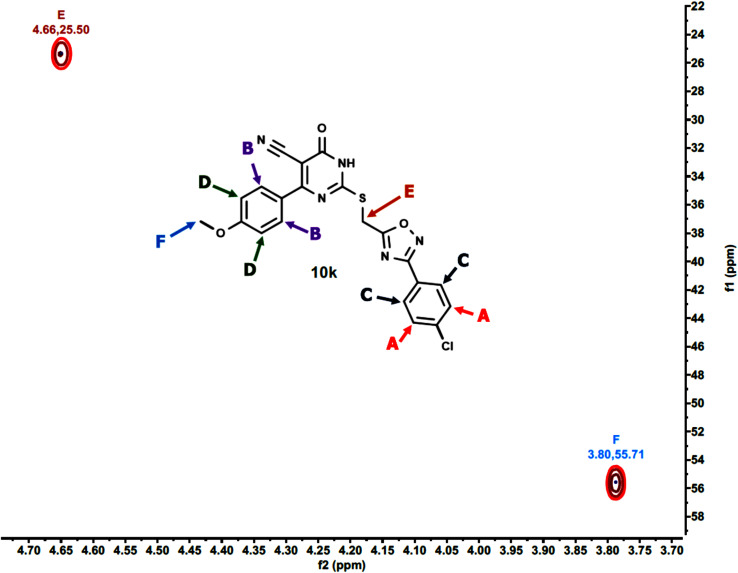
Expanded HSQC-2D NMR of 10k.

**Fig. 7:**
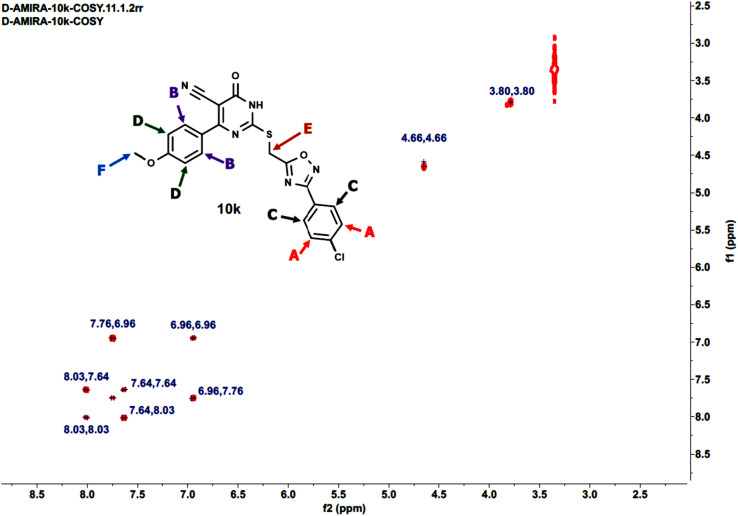
COSY-2D NMR of 10k.

**Fig. 8:**
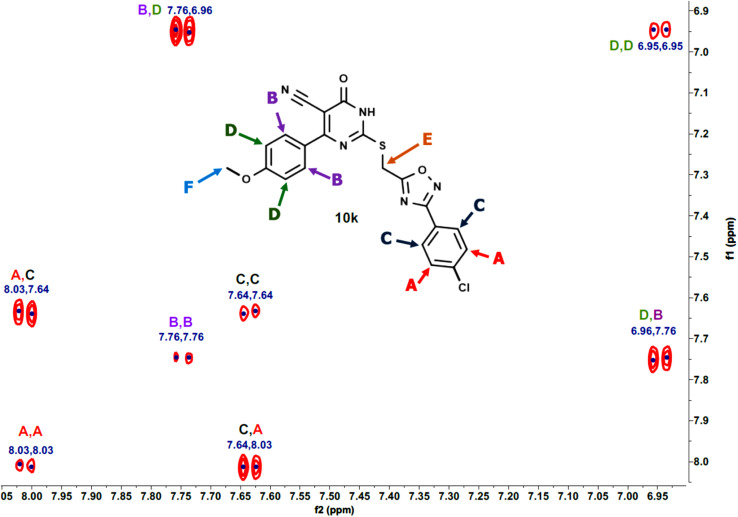
Expanded COSY-2D NMR of 10k.

Furthermore, the 2D NMR investigations (HSQC and COSY) satisfactorily confirmed the chemical structures of compounds 10k and 10a, which served as illustrative examples. The spectra reveal clear correlations between protons and their connected carbons, as well as connectivity among surrounding protons. For example, the spectra for compound 10k clearly resolve all of the molecule's protonated carbons, validating the given structure (see Table 1 and Fig. 4–8).

**Table 1:** The HSQC spectrum (C–H correlations) of compound 10k

Label	*δ* ^1^H(ppm)	*δ* ^13^C (ppm)	Assignment
A	8.03	129.30	Aromatic C–H (chlorophenyl ring)
B	7.76	130.18	Aromatic C–H (pyrimidinone ring)
C	7.64	129.91	Aromatic C–H (chlorophenyl ring)
D	6.96	113.85	Aromatic C–H (methoxyphenyl ring)
E	4.66	25.50	Methylene linker (S–CH_2_)
F	3.80	55.71	Methoxy group (OCH_3_)

The COSY spectrum of 10k supports the spatial correlations between these protons: A–C correlation: A clear cross-peak between protons A (8.03 ppm) and C (7.64 ppm) reveals they are connected on the same aromatic ring (chlorophenyl ring), as illustrated in Fig. 8 and B–D correlation: On the methoxyphenyl ring, there is a cross-peak between proton B (7.76 ppm) and proton D (6.96 ppm). This confirms the two ring systems' connection and orientation (*via* the *p*-disubstitution pattern).

**Fig. 9:**
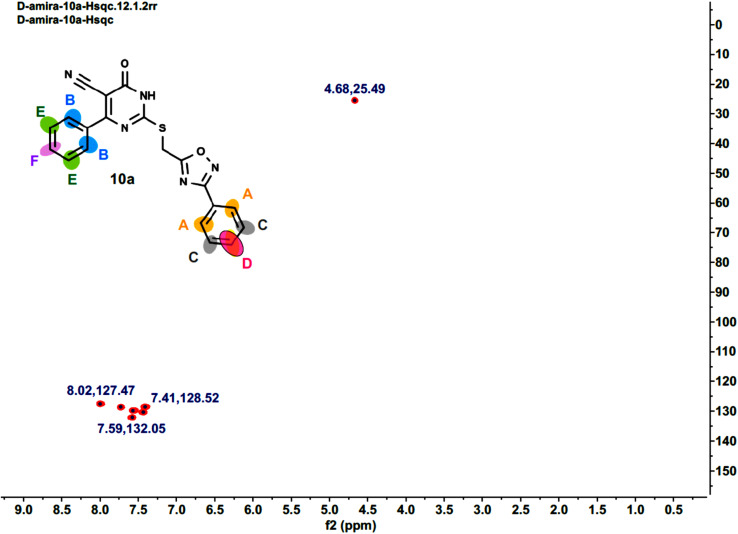
HSQC-2D NMR of 10a.

**Fig. 10:**
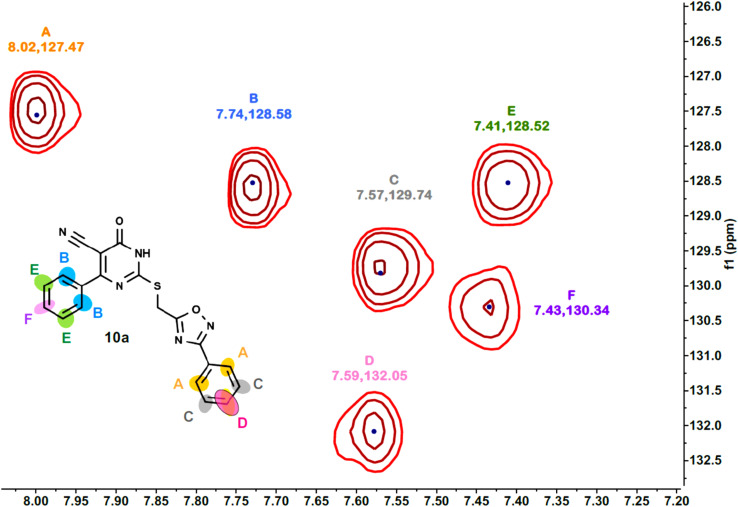
Expanded HSQC-2D NMR of 10a.

**Fig. 11:**
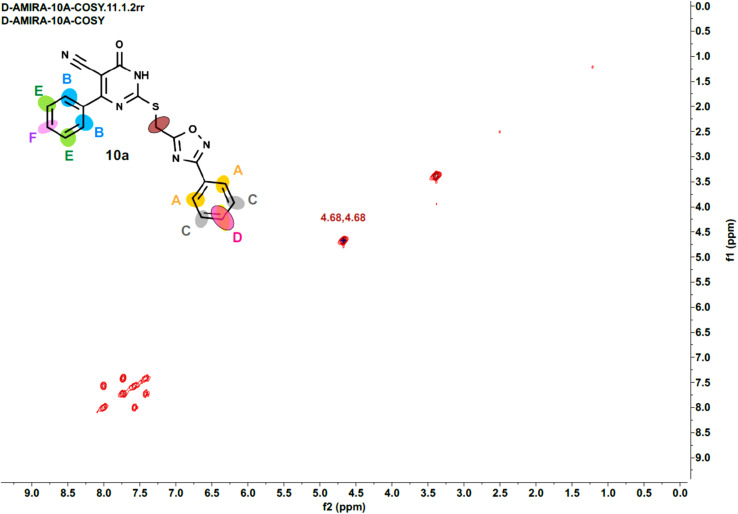
COSY-2D NMR of 10a.

**Fig. 12:**
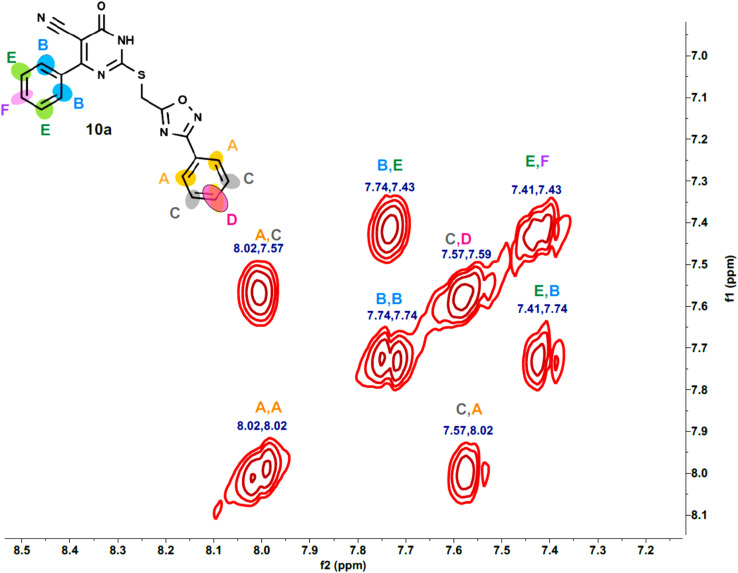
Expanded COSY-2D NMR of 10a.

The spectra for compound 10a are more complex due to overlapping signals in the aromatic region. However, the 2D data allows for a complete assignment, as indicated Table 2 and Fig. 9–12.

**Table 2:** The HSQC spectrum of compound 10a

Label	*δ* ^1^H(ppm)	*δ* ^13^C (ppm)	Assignment
A	08.02	127.47	Aromatic C–H (phenyl ring)
B	7.74	128.58	Aromatic C–H (phenyl ring)
C	7.57	129.74	Aromatic C–H (phenyl ring)
D	7.59	132.05	Aromatic C–H (phenyl ring)
E	7.41	128.52	Aromatic C–H (phenyl ring)
F	7.43	130.34	Aromatic C–H (phenyl ring)
Linker	4.68	25.49	Methylene linker (S–CH_2_-Oxadiazole)

The COSY spectrum is essential for separating the packed aromatic signals into two separate groups: Spin System 1 (Right-side ring): Cross-peaks link protons A (the most down fielded proton next to the withdrawing oxadiazole ring) to C and C to D. This demonstrates that these three protons are all neighbors on the same aromatic ring, and there is no association between them and the other three protons B (next to the pyrimidinone oxadiazole ring), E, and F. Spin System 2 (Left-side ring): Cross-peaks connect protons B to E and E to F belong to another aromatic distinct spin system, as depicted in Fig. 12.

### 3.2. Biology

#### 3.2.1. Cell viability assay

The effect of new compounds 10a–l on the viability of a normal human cell line, namely human mammary gland epithelial cells (MCF-10A), was evaluated to determine the safety of the newly synthesized compounds. The MTT assay was utilized to evaluate the cell viability of the new compounds following a four-day incubation with MCF-10A cells.^29,37^Table 3 demonstrates that none of the examined compounds displayed cytotoxicity towards normal cells; all compounds maintained cell viability over 89% at a dose of 50 μM.

**Table 3:** Cell viability assay and IC_50_ values of compounds 10a–l against four cancer cell lines^a^

Comp	Ar	X	Cell viability%	Antiproliferative activity IC_50_ ± SEM (nM)				
				A-549	MCF-7	Panc-1	HT-29	Average (GI_50_)
10a	Phenyl	H	91	44 ± 3	40 ± 3	42 ± 3	45 ± 3	43
10b	Phenyl	Cl	90	65 ± 5	61 ± 5	61 ± 5	66 ± 5	63
10c	Phenyl	OMe	92	49 ± 3	44 ± 3	44 ± 3	51 ± 3	47
10d	4-Pyridyl	H	89	56 ± 4	51 ± 4	53 ± 4	57 ± 4	54
10e	4-Pyridyl	Cl	90	30 ± 2	25 ± 1	28 ± 1	30 ± 2	28
10f	4-Pyridyl	OMe	91	52 ± 4	49 ± 3	50 ± 4	54 ± 4	51
10g	4-Chlorophenyl	H	90	39 ± 2	35 ± 2	37 ± 2	40 ± 2	38
10h	4-Chlorophenyl	Cl	92	35 ± 2	31 ± 2	35 ± 2	36 ± 2	34
10i	4-Chlorophenyl	OMe	91	33 ± 2	28 ± 1	30 ± 1	34 ± 1	31
10j	4-Methoxyphenyl	H	89	69 ± 5	65 ± 5	68 ± 5	70 ± 5	68
10k	4-Methoxyphenyl	Cl	90	23 ± 1	20 ± 1	22 ± 1	23 ± 1	22
10l	4-Methoxyphenyl	OMe	91	28 ± 1	22 ± 1	26 ± 1	28 ± 1	26
Erlotinib	—	—	ND	30 ± 3	40 ± 3	30 ± 3	30 ± 3	33

a —: Not applicable, ND: not determined.

#### 3.2.2. Antiproliferative assay

The MTT assay^43^ was utilized to assess the antiproliferative effects of compounds 10a–l on four human cancer cell lines: HT-29 (colon cancer), Panc-1 (pancreatic cancer), A-549 (lung cancer), and MCF-7 (breast cancer). Erlotinib served as a reference.^38,39^Table 3 presents the median inhibitory concentration (IC_50_) and average IC_50_ (GI_50_) values for each compound evaluated across the four cancer cell lines.

Compounds 10a–l exhibited potent antiproliferative activity, with GI_50_ values between 22 and 68 nM, in comparison to the reference erlotinib (GI_50_ = 33 nM). Moreover, all assessed compounds exhibited superior affinity for the breast (MCF-7) and pancreatic (Panc-1) cancer cell lines compared to the other cell lines investigated. Compounds 10e, 10h, 10i, 10k, and 10l exhibited the highest antiproliferative activity, with GI_50_ values of 28, 31, 34, 22, and 26 nM, respectively. Compounds 10e, 10i, 10h, 10k, and 10l all outperformed erlotinib against the breast cancer (MCF-7) cell line, with IC_50_ values ranging from 20 to 31 nM, compared to 40 nM for erlotinib. Moreover, derivatives 10e, 10k, and 10l showed superior efficacy compared to erlotinib against the Panc-1 (pancreatic) cancer cell line. Their IC_50_ values were 28, 22, and 26 nM, respectively, while erlotinib exhibited an IC_50_ value of 30 nM.

Compound 10k (Ar = 4-OMe-Ph, R = Cl) surpassed all other examined compounds. It exhibited a GI_50_ of 22 nM, rendering it 1.5 times more potent than erlotinib (GI_50_ = 33 nM) against the four cancer cell lines examined. Compound 10k exhibited potent antiproliferative activity against the breast (MCF-7) cancer cell line, with an IC_50_ value of 20 nM, which is twice as potent as erlotinib's IC_50_ value of 40 nM. Moreover, compound 10k demonstrates a 1.3-fold greater potency than erlotinib against the other three cell lines, as shown in Table 3.

The substitution pattern at position six significantly impacts the antiproliferative efficacy of compounds 10a–l (aryl group) of the pyrimidine moiety and position four of the phenyl group in the 1,2,4-oxadiazole moiety. For example, compound 10b (Ar = Ph, R = Cl), a derivative with a phenyl group attached to the sixth position of the pyrimidine moiety, demonstrated inferior efficacy as an antiproliferative agent compared to the *p*-methoxyphenyl derivative, 10k (Ar = 4-OMe-Ph, R = Cl). Compound 10b exhibited a GI_50_ of 63 nM, indicating a potency that is 2.9-fold lower than that of 10k, illustrating that the *p*-methoxyphenyl group at the 6-position of the pyrimidine moiety is more conducive to antiproliferative activity than the unsubstituted phenyl group.

The substitution of the *p*-methoxyphenyl group at the 6-position of the pyrimidine moiety with different aryl groups led to a moderate to significant reduction in antiproliferative efficacy. Compounds 10e (Ar = pyridin-3-yl, R = Cl) and 10h (Ar = 4-chlorophenyl, R = Cl), which are derivatives containing pyridine and 4-chlorophenyl, demonstrated reduced potency compared to 10k. Compounds 10e and 10h demonstrated IC_50_ values of 28 and 34 nM, respectively. The compounds demonstrated reductions in potency of 1.3- and 1.6-fold relative to compound 10k, indicating that the *p*-methoxyphenyl and pyridyl moieties are more tolerated than the 4-chlorophenyl moiety for antiproliferative action.

Additionally, the substitution pattern at the *para* position of the phenyl group within the 1,2,4-oxadiazole moiety may significantly influence the antiproliferative action of compounds 10a–l. Compounds 10j (Ar = 4-OMe-Ph, R = H) and 10l (Ar = 4-OMe-Ph, R = OMe), which have the same structural characteristics as 10k but with an unsubstituted phenyl group as in 10j or a methoxy derivative as in 10l, had IC_50_ values of 68 nM and 26 nM, respectively. Compound 10j demonstrated a GI_50_ value of 68 nM, rendering it 3-fold less efficient than 10k and the least potent derivative among the newly synthesized compounds. These data indicated that an unsubstituted phenyl group within the 1,2,4-oxadiazole moiety is not favorable to antiproliferative activity. Compound 10l, a methoxyphenyl derivative within the 1,2,4-oxadizole moiety, had a GI_50_ value of 26 nM, which was slightly less potent than the *p*-chloro derivative, 10K (GI_50_ = 22 nM), indicating that substitution with either an electron withdrawing group (chlorine atom) or an electro donating group (methoxy group) is beneficial for the antiproliferative action, with the chlorine atom having higher activity.

#### 3.2.3. EGFR inhibitory assay

The most effective antiproliferative derivatives, 10e, 10i, 10h, 10k, and 10l, were assessed for their ability to inhibit EGFR using the EGFR-TK test. The results are presented in Table 4. Erlotinib served as the reference compound.^33,40^

**Table 4:** IC_50_ values of compounds 10e, 10i, 10h, 10k, and 10l against EGFR and VEGFR-2

Compound	EGFR inhibition IC_50_ ± SEM (nM)	VEGFR-2 inhibition IC_50_ ± SEM (nM)
10e	65 ± 4	32 ± 2
10h	79 ± 5	43 ± 3
10i	71 ± 5	39 ± 2
10k	57 ± 3	21 ± 1
10l	61 ± 4	26 ± 1
Erlotinib	80 ± 5	—
Sorafenib	—	0.17 ± 0.001

The findings of this assay align with those of the antiproliferative assay, indicating that compounds 10k (Ar = 4-OMe-Ph, R = Cl) and 10l (Ar = 4-OMe-Ph, R = 4-OMe), identified as the most potent antiproliferative agents, were the most efficacious derivatives of EGFR inhibitors, exhibiting IC_50_ values of 57 ± 3 and 61 ± 4 nM, respectively. They exhibited 1.4- and 1.3-fold more potency than erlotinib (IC_50_ = 80 nM). Furthermore, compounds 10e (Ar = pyridin-3-yl, R = Cl) and 10i (Ar = 4-chlorophenyl, R = OMe) have substantial EGFR inhibitory activity, with IC_50_ values of 65 and 71 nM, respectively, which were marginally more potent than the reference erlotinib. Ultimately, compound 10h (Ar = 4-chlorophenyl, R = Cl) demonstrated equivalent EGFR inhibitory action to erlotinib, with an IC_50_ value of 79 nM. The data from these *in vitro* experiments indicated that compounds 10e, 10i, 10k, and 10l were effective antiproliferative agents potentially functioning as EGFR inhibitors.

#### 3.2.4. VEGFR-2 inhibitory assay

Using sorafenib as a reference, the most potent antiproliferative derivatives (10e, 10i, 10h, 10k, and 10l) were tested for their capacity to inhibit VEGFR-2.^28,35^ The results are shown in Table 4 as IC_50_ values. All data are the average of three experiments ± SD. The evaluated derivatives demonstrated substantial VEGFR-2 inhibitory activity, with IC_50_ values ranging from 21 nM to 43 nM. In all instances, the examined compounds exhibited elevated IC_50_ values (indicating reduced potency) compared to the reference medication sorafenib (IC_50_ = 0.17 nM).

The findings from this *in vitro* assay align with the results of both antiproliferative and EGFR inhibitory assays, indicating that compounds 10k and 10l, the most effective antiproliferative and EGFR inhibitors, are also the most potent derivatives as VEGFR-2 inhibitors, exhibiting IC_50_ values of 21 and 26 nM, respectively, compared to sorafenib, which displayed an IC_50_ value of 0.17 nM. Compound 10e exhibited the third–highest activity, with an IC_50_ value of 32 nM. Finally, compounds 10h and 10i exhibited the lowest potency as VEGFR-2 inhibitors, with IC_50_ values of 43 and 39 nM, respectively. These results indicate that compounds 10k and 10l are effective antiproliferative candidates that may act as dual inhibitors of EGFR and VEGFR-2.

#### 3.2.5. Apoptotic markers assays

Dysregulation of apoptosis is a characteristic feature of human cancer, leading to unregulated proliferation, inadequate treatment responses, and the emergence of drug-resistant cells.^44^ Consequently, contemporary anticancer medicines are acknowledged for their capacity to trigger apoptosis in cancer cells through both extrinsic and intrinsic pathways.^41,45^ The impact of compounds 10k and 10l, the most potent derivatives in all *in vitro* assays, on caspase-3 was assessed employing the MCF-7 breast cancer cell line and compared to staurosporine as a reference medication (Table 5). The investigation revealed that 10k was the most effective derivative, showing a significant rise in caspase-3 protein levels (590 ± 5 pg mL^−1^) compared to the reference staurosporine (465 ± 4 pg mL^−1^). Compound 10k increased active caspase-3 levels by 9-fold when compared to control untreated MCF-7 cells, and induced caspase-3 levels were higher than those produced by staurosporine. Compound 10l elevated active caspase-3 levels by 8-fold (530 ± 4 pg mL^−1^) relative to untreated MCF-7 cells, as illustrated in Table 5. Although compound 10l elevates caspase-3 levels less than compound 10k, it remains more effective than the standard staurosporine.

**Table 5:** Caspases 3, 8, and 9 levels for compounds 10k and 10l

Compd. no.	Caspase-3		Caspase-8		Caspase-9	
	Conc (pg ml^−1^)	Fold change	Conc (ng ml^−1^)	Fold change	Conc (ng ml^−1^)	Fold change
10k	590 ± 5	9.00	2.50 ± 0.20	25.00	24 ± 1	24
10l	530 ± 4	8.00	2.00 ± 0.10	20.00	22 ± 1	22
Staurosporine	465 ± 4	7.00	1.90 ± 0.10	19.00	20 ± 1	20
Control	65	1.0	0.10	1	1	1

To elucidate the apoptotic processes of compounds 10k and 10l, whether *via* the intrinsic or extrinsic pathway, their impacts on caspase-8 and caspase-9 were evaluated. The results indicated that compound 10k increases the levels of caspase-8 and caspase-9 by 25 and 24-fold, respectively, whereas compound 10l elevates the levels of caspase-8 and caspase-9 by 20 and 22-fold, respectively, compared to the control MCF-7 cancer cells. This signifies the activation of both intrinsic and extrinsic pathways (Table 5).

The Bcl-2 protein family, which includes pro-apoptotic proteins (Bax) and anti-apoptotic proteins (Bcl-2), primarily regulates apoptosis.^46^ Various studies have demonstrated a substantial correlation between elevated Bcl-2 levels and reduced Bax levels, which are associated with tumor cell proliferation.^47,48^ Consequently, we assessed the expression levels of Bcl-2 and Bax proteins in MCF-7 breast cancer cells treated with compounds 10k and 10l, Table 6.

**Table 6:** Apoptotic markers assays for 10k and 10l in breast (MCF-7) cancer cell line

Compound no.	Bcl-2 (ng mL^−1^)	Fold reduction	Bax (pg mL^−1^)	Fold change	p53 (pg mL^−1^)	Fold change
10k	1.20 ± 0.001	4	545 ± 3	9	330 ± 2	5
10l	1.50 ± 0.001	3	505 ± 3	8	285 ± 2	4
Control	5	1	60	1	65	1

Table 6 demonstrates that compound 10k resulted in a 9-fold elevation in Bax levels and a 5-fold reduction in Bcl-2 levels compared to control, untreated cells. Furthermore, compound 10l exhibited an 8-fold increase in Bax levels and a 4-fold decrease in Bcl-2 levels. These observations indicate that apoptosis may contribute to the antiproliferative effects of the examined compounds.

P53's ability to eliminate superfluous, damaged, or contaminated cells by apoptosis is critical for the proper regulation of cell proliferation in multicellular organisms.^49^ p53 is activated by both external and internal stress signals, allowing for nuclear accumulation in an active state. As a result, p53 induces either reversible cell growth arrest or apoptosis. The aforesaid activity is necessary for tumor suppression.^50^ The p53 levels in breast (MCF-7) cancer cells treated with compounds 10k and 10l increased significantly, exceeding those in untreated control cells by at least fourfold. This finding shows that high levels of the p53 protein may control the apoptotic process in these new compounds.

#### 3.2.6. Cytotoxicity against normal cell line

To determine the selectivity of the target compounds for cancer cells against normal cells, the safety profiles of the two most potent compounds, 10k and 10l, were evaluated using the MTT assay on the normal human diploid cell line (WI-38). The IC_50_ values for the investigated compounds 10k and 10l exceeded 200 nM. Table 7 demonstrates that the evaluated substances had an improved safety margin concerning normal cells.

**Table 7:** Selectivity index of compounds 10k and 10l

Compound	Cytotoxicity (WI-38) IC_50_ (nM)	Selectivity index (SI)			
		A-549	MCF-7	Panc-1	HT-29
10k	> 200	> 8.0	> 10.0	> 9.0	> 8.0
10l	> 200	> 7.0	> 9.0	> 7.0	> 7.0

#### 3.2.7. Antioxidant activity

Redox homeostasis is crucial for biological function, and its disruption results in significant pathophysiological effects in cells, highlighting the equilibrium between the levels of reactive oxygen species (ROS) and antioxidants.^51,52^ Cells may produce excessive reactive oxygen species (ROS) as an inevitable consequence of modifications in metabolic signaling pathways.^53^ Excessive levels of ROS beyond non-toxic thresholds can induce oxidative damage to macromolecules, including nucleic acids, proteins, lipids, and glucose, leading to enzyme fragmentation, structural protein degradation, membrane impairment, gene mutations, and the activation of pro-oncogenic signaling pathways.^54^ Elevated oxidative stress can trigger tumorigenesis and facilitate tumor progression by directly oxidizing macromolecules or through aberrant redox signaling induced by oxidative stress, indicating that elevated ROS levels may heighten cancer risk when antioxidant defenses are inadequate to safeguard cells from oxidative stress.^55,56^ Because oxidative stress plays a significant role in carcinogenesis and cancer progression, using antioxidants to treat cancer is an appealing concept.^57–59^

Trolox was used as a control to evaluate the potential antioxidant properties of compounds 10k and 10l using the scavenging of stable free radicals by 2,2-diphenyl-1-picrylhydrazyl (DPPH).^42,60,61^ Three different concentrations of the compounds under investigation (100 μM, 50 μM, and 10 μM) were used for the experiment. Table 8 presents the findings.

**Table 8:** Antioxidant activity of compounds 10k and 10l

Antioxidant (DPPH radical scavenging activity %)			
Comp	100 μM	50 μM	10 μM
10k	94.5	81.9	72.5
10l	92.7	80.6	69.8
Trolox	95.2	82.5	77.6

Compared to trolox (77.6%), compounds 10k and 10l showed significant antioxidant activity at 10 μM, with DPPH radical scavenging of 72.5% and 69.8%, respectively. As seen in Table 6, compounds 10k and 10l had comparable radical scavenging activity to trolox at doses of 100 and 50 μM, respectively. The data suggest that compounds 10k and 10l may be classified as effective antiproliferative agents possessing antioxidant properties.

### 3.3. Computational approaches

#### 3.3.1. Molecular mechanics-based simulations

To further validate and explore the conformational behavior and non-covalent interaction profiles of these compounds within the kinase binding pockets, molecular mechanics (MM) simulations were employed in the active sites of both EGFR and VEGFR-2.^62^ These classical physics-based methods allowed for the evaluation of energy-minimized binding poses and the prediction of how structural variations influence molecular recognition.^63^ Compounds 10k, and 10i characterized by a 4-methoxyphenyl substituent, consistently adopted a low-energy conformation with optimal orientation in both kinases, engaging in key hydrogen bonding and hydrophobic interactions that stabilized its binding. In contrast, the unsubstituted phenyl group in 10b contributed to a less favorable fit and weaker overall binding interactions with EGFR and VEGFR-2. The MM-derived potential energy surfaces and interaction profiles highlighted the energetic contribution of key functional groups, particularly the methoxy group in 10k, and 10i to enhanced ligand–receptor affinity. These insights provided a structural explanation for the superior antiproliferative activity of 10k, and 10i as observed experimentally. By capturing the energetic preferences and spatial complementarity of these compounds, MM simulations supported the observed activity trends and offered predictive insights into how specific molecular features influence binding performance.^64^ These findings establish a foundation for the rational design of next-generation inhibitors with improved selectivity and potency toward EGFR and VEGFR-2.^65^

##### 3.3.1.1. Molecular docking simulations

To investigate the molecular basis of kinase inhibition by the newly synthesized compounds, docking studies were performed for 10k, and 10i the most potent antiproliferative agents, and 10b, a less active analogue, against EGFR (PDB ID: 1M17). Additionally, our compounds were evaluated for its binding interactions with VEGFR-2 (PDB ID: 3WZE).^35,66^ The FDA-approved inhibitors erlotinib and sorafenib were used as reference ligands for EGFR and VEGFR-2, respectively. Protein structures were obtained from the Protein Data Bank and prepared using the CDOCKER module in Discovery Studio 2016, applying the CHARMm force field.^67^ Prior to docking, all heteroatoms and solvent molecules within 5 Å of the co-crystallized ligand were removed. Hydrogen atoms were added, and the protonation states of titratable residues were adjusted to reflect physiological conditions (pH 7.4), including careful assignment of histidine tautomeric forms.

The receptor structures were subjected to energy minimization until reaching a convergence threshold of 0.01 kcal mol^−1^ Å RMS gradient, ensuring an energetically favorable yet structurally preserved active site. Ligands were prepared using the “Prepare Ligands” protocol, including 3D geometry correction, stereoisomer generation, and assignment of appropriate protonation states. A rigid receptor–flexible ligand approach was adopted, enabling full conformational sampling of the ligand torsions while maintaining a static protein backbone. Docking grids were centered on the coordinates of the native co-crystallized inhibitors to ensure accurate sampling of the kinase ATP-binding sites.^68,69^ For each ligand, ten poses were generated. The pose with the most favorable CDOCKER interaction energy, accounting for both van der Waals and electrostatic contributions, was selected for detailed analysis.

**Fig. 13:**
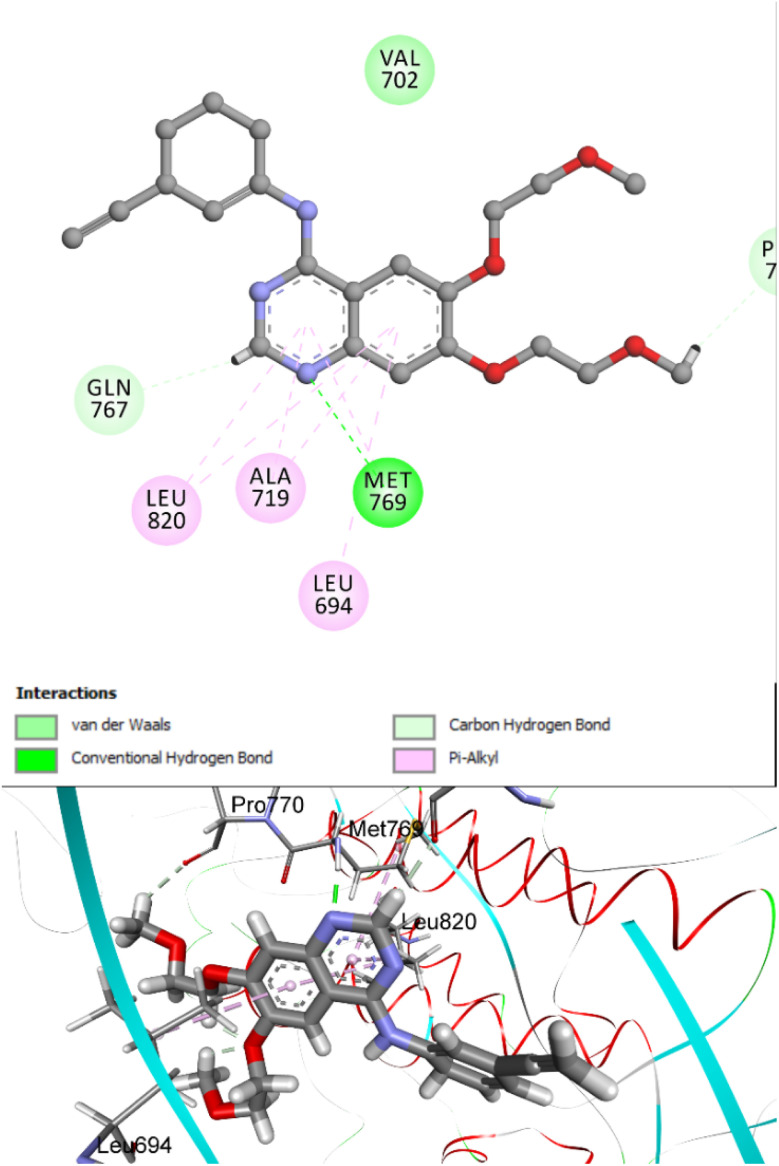
Superimposed pose of erlotinib re-docked into the EGFR active site (PDB ID: 1M17). The predicted conformation shows close overlap with the crystallographic orientation (RMSD = 1.26 Å), preserving key interactions such as the hydrogen bond with Met769. This validates the accuracy of the docking workflow used in this study.

To confirm the reliability of the docking protocol, self-docking validation was conducted by reintroducing the native ligand into its crystallographic site. The resulting root mean square deviation (RMSD) values were within acceptable limits, supporting the accuracy of the docking setup. The reliability of the docking protocol was verified through self-docking validation, where the native ligand (erlotinib) was re-docked into its original EGFR binding site. The resulting RMSD of 1.26 Å and a re-docking score of −7.97 kcal mol^−1^ demonstrated a strong alignment with the experimentally observed conformation, confirming the robustness and predictive accuracy of the docking workflow. Notably, the canonical hinge-region hydrogen bond between the pyrimidine nitrogen of the docked ligands and Met769 of EGFR was consistently reproduced, underscoring its essential role in stabilizing the compounds within the ATP-binding pocket (Fig. 13).

**Fig. 14:**
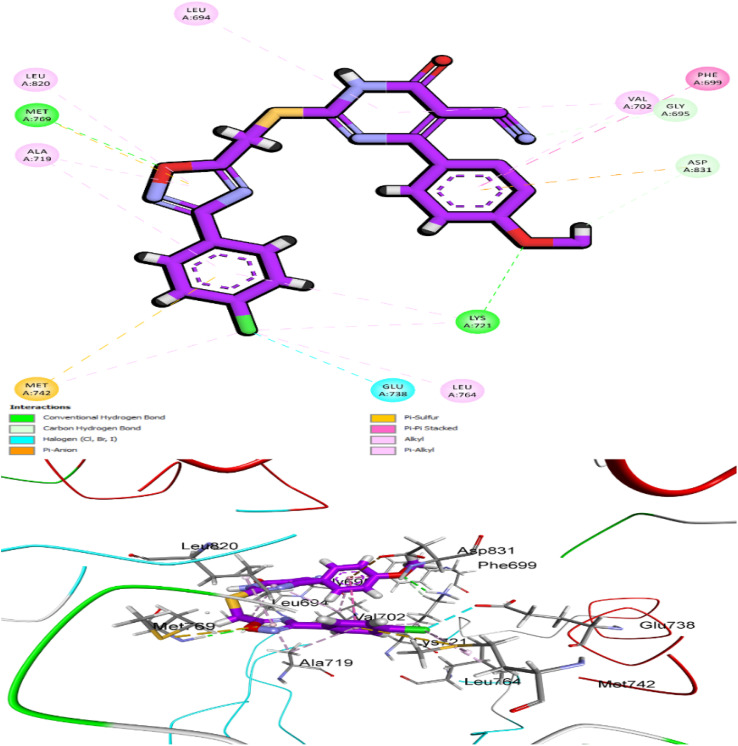
Top: 2D interaction map showing 10k docked into the EGFR active site (PDB ID: 1M17). Key contacts include a hinge-region hydrogen bond with Met769 and π–π stacking with Phe699. The methoxy-substituted phenyl group maintains oxadiazole planarity between critical residues. Bottom: 3D view of the binding pose within the kinase cleft, highlighting spatial complementarity and residue engagement.

Docking simulations of 10k revealed a highly favorable binding pose within the ATP-binding pocket of EGFR, supported by a CDOCKER interaction energy of −8.21 kcal mol^−1^ and an RMSD of 1.17 Å. These computational metrics are consistent with the compound's superior *in vitro* potency (IC_50_ = 57 ± 3 nM) compared to the reference drug erlotinib (IC_50_ = 80 ± 5 nM). Visual analysis of the docked complex confirmed a snug fit of 10k within the active site, with all key structural components engaging in specific and complementary interactions with critical kinase residues (Fig. 14).

Compound 10k exhibits a well-defined pharmacophoric architecture composed of two key structural elements: core (1,2,4-oxadiazole ring and dihydropyrimidine-5-carbonitrile ring), and terminal phenyl rings (4-methoxyphenyl & *para*-chlorophenyl). Each of these features contributes uniquely to the high binding affinity within the ATP-binding pocket of EGFR. The 1,2,4-oxadiazole ring serves as the central anchoring motif, engaging in a crucial hydrogen bond with the hinge residue Met769, a conserved interaction essential for effective ATP-competitive kinase inhibition. This ring is maintained in a planar conformation, which is critical for its proper alignment within the narrow hinge region. Its planarity is reinforced by the adjacent 4-methoxyphenyl group, which stabilizes the orientation of the oxadiazole through both electronic and steric effects. Also, the 1,2,4-oxadiazole ring engages in π–π stacking with Ala719 and Leu820. The methoxy substituent not only promotes this planar geometry but also contributes to hydrophobic and π–π stacking interactions with Phe699, and forms π–alkyl contacts with Val702. Adjacent to the oxadiazole, the dihydropyrimidine-5-carbonitrile core occupies the central polar region of the EGFR binding pocket. This scaffold contributes to molecular recognition through hydrogen bonding and electrostatic interactions, primarily *via* its cyano group, which interact favorably with residue Asp831, enhancing binding stability and directional specificity. The ring is oriented toward hydrophobic residues such as Leu694, participating in π–alkyl contact.

Completing the pharmacophore is the *para*-chlorophenyl moiety, which projects into a hydrophobic pocket near the gatekeeper region. The chlorine atom facilitates van der Waals and halogen bonding interactions with residues including Leu764 and Glu738, helping to fill the lateral volume of the binding site and further anchoring the ligand through nonpolar stabilization. Together, these four pharmacophoric elements work in concert to establish a dense and well-balanced network of interactions within the EGFR active site. Their combined effects explain the strong binding affinity observed in docking simulations and align with the potent *in vitro* inhibitory activity demonstrated by compound 10k.

**Fig. 15:**
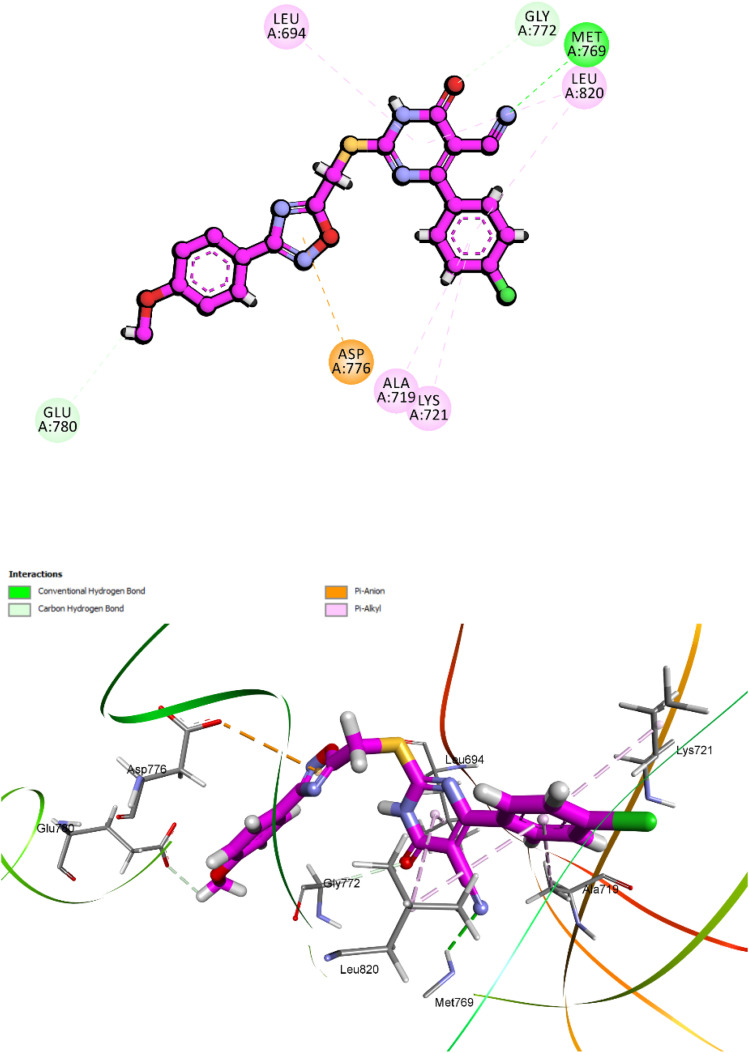
5Molecular docking pose of compound 10i within the active site of EGFR.

Compound 10i, a closely related analog of 10k, was also subjected to docking analysis (Fig. 15). Interestingly, while 10i shares the same pharmacophoric elements, the substitution pattern differs. In 10i, the 4-methoxyphenyl group is attached to the oxadiazole ring, while the *para*-chlorophenyl group is connected to the dihydropyrimidine-5-carbonitrile scaffold, thus reversing the orientation relative to compound 10k. Despite this inversion, 10i demonstrated a highly favorable binding conformation with EGFR, reflected by a CDOCKER score of −8.19 kcal mol^−1^ and an RMSD of 1.24 Å. Its interaction profile mirrors many of the critical contacts seen in 10k. The cyano group of the dihydropyrimidine-5-carbonitrile ring forms a crucial hydrogen bond with Met769, the *para*-chlorophenyl group engages in π–alkyl contacts with Leu820, Lys721, and Ala719, the oxadiazole moiety maintains a π–anion interaction with Asp776, and the methoxy substituent contributes an additional C–H interaction with Glu780. These complementary contacts reinforce the stable binding of 10i and explain its potent *in vitro* inhibitory activity, thereby highlighting its adaptability within the EGFR pocket while maintaining the hallmark hinge-binding motif.

**Fig. 16:**
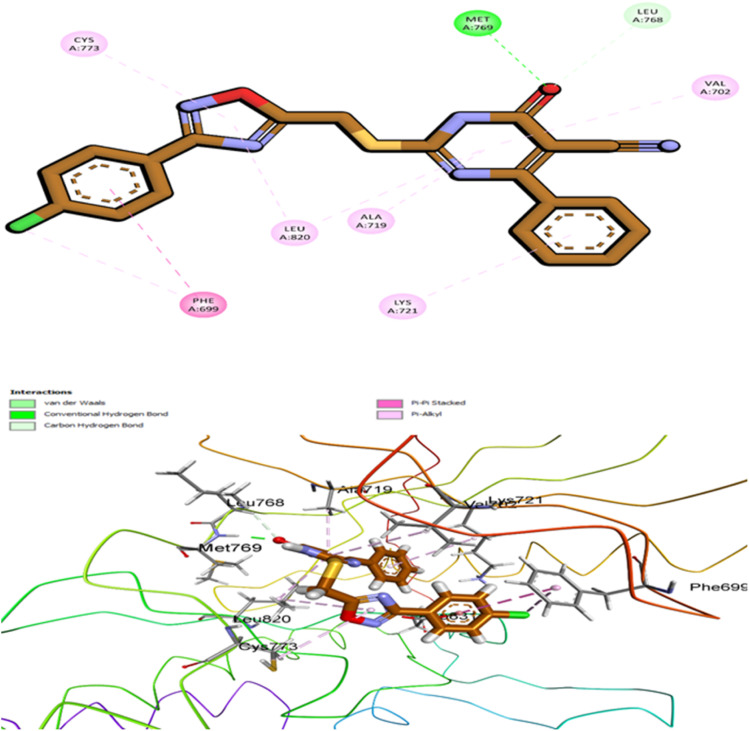
Top: 2D interaction diagram of compound 10b bound to EGFR (PDB ID: 1M17), showing weak bonding and limited hydrophobic contacts. Bottom: 3D view of the docked conformation, highlighting the misaligned oxadiazole ring and minimal engagement with the hinge and hydrophobic regions.

To complement the experimental findings, compound 10b was subjected to molecular docking analysis against the EGFR kinase domain (PDB ID: 1M17) to evaluate its binding pose and interaction profile. The docking results revealed a CDOCKER interaction energy score of −6.31 kcal mol^−1^ and an RMSD of 1.91 Å, indicating a weaker binding affinity and less stable conformation compared to the more active analog 10k. These observations are consistent with lower *in vitro* inhibitory activity (GI_50_ = 63 nM) of 10b and confirm its suboptimal engagement within the receptor binding cleft (Fig. 16).

Structurally, compound 10b preserves the same fundamental pharmacophoric elements as 10k and 10i: a core (1,2,4-oxadiazole ring and dihydropyrimidine-5-carbonitrile ring), and terminal phenyl rings (phenyl & *para*-chlorophenyl). However, the nature of the substituents particularly the lack of a methoxy group on the first ring critically impairs the orientation and interaction potential. The 1,2,4-oxadiazole ring, while still present, fails to adopt a favorable planar conformation between the hinge residues. In contrast to 10k, and 10i where the methoxy-substituted phenyl ring enforced rigidity and alignment, the unsubstituted phenyl ring in 10b lacks the electronic and steric support needed to stabilize the oxadiazole ring in the correct orientation. As a result, the oxadiazole is partially misaligned and cannot effectively position its ring for network bonding. The dihydropyrimidine-5-carbonitrile core in 10b continues to provide some polar functionality, contributing carbonyl-interaction with Met769.

The geometry of the entire scaffold is less compact and deviates from the ideal alignment seen in high-affinity inhibitors. The terminal phenyl ring, in place of the methoxyphenyl found in 10k, and 10i lacks both the electron-donating effect and hydrophobic bulk that promote favorable pocket filling and aromatic interactions. Consequently, this ring establish π–alkyl interactions with residue Lys721. The *para*-chlorophenyl moiety, although retained from 10k, and 10i plays a limited role in stabilizing the ligand. While some weak π-π contact with Phe699 is observed, the absence of strong complementary interactions results in insufficient binding stabilization.

In summary, compound 10b exhibits a pharmacophore arrangement similar to that of 10k but lacks the conformational integrity and electronic enhancements required for effective binding. The failure of the phenyl ring to maintain the planarity of the oxadiazole ring disrupts critical hinge-region interactions and leads to reduced engagement with key residues across the EGFR active site. These structural deficiencies account for its diminished docking performance and correspond well with its lower experimental potency.

**Fig. 17:**
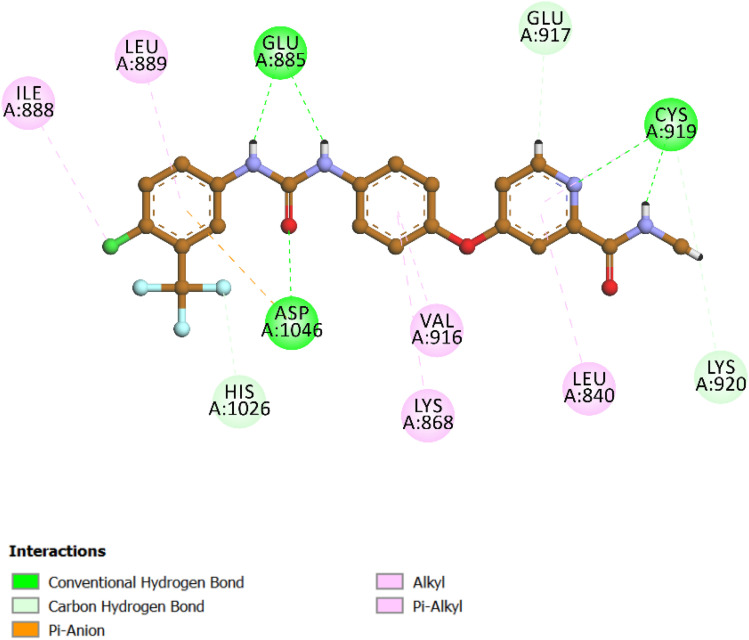
Predicted binding pose and 2D interaction map of the reference ligand sorafenib within the VEGFR-2 active site (PDB ID: 3WZE). The docking pose demonstrates excellent agreement with the experimental geometry (RMSD = 1.25 Å) and a strong binding affinity (CDOCKER interaction energy = −8.47 kcal mol^−1^).

To deepen the structural understanding of ligand–receptor recognition within the VEGFR-2 kinase domain, molecular docking simulations were performed using PDB ID: 3WZE. The clinically approved VEGFR-2 inhibitor sorafenib was employed as a reference ligand to validate the docking protocol and establish a performance benchmark for the synthesized analogs. The docking output revealed a CDOCKER interaction energy score of −8.47 kcal mol^−1^ and an RMSD of 1.25 Å upon re-docking, indicating strong agreement with the experimentally observed binding mode and confirming the reliability of the computational workflow (Fig. 17).

Interaction profiling of the docked pose demonstrated that sorafenib maintained a stable and highly specific network of interactions within the ATP-binding cleft of VEGFR-2. Key conventional hydrogen bonds were observed with critical residues including Glu885, Cys919, and Asp1046, which are well-documented as essential anchors for potent ATP-competitive kinase inhibitors. These polar interactions formed the core of the binding stability. In addition to hydrogen bonding, sorafenib established a robust array of hydrophobic interactions notably pi–alkyl and alkyl contacts with residues such as Val916, Leu840, Lys868, and Leu889. These hydrophobic contacts further stabilized the ligand within the binding cavity and contributed to the optimal occupation of the non-polar subpocket.

Of particular significance was a pi–anion interaction with Asp1046, enhancing the electrostatic complementarity of the ligand and reinforcing its high binding affinity. Together, these findings confirm that the docking protocol accurately recapitulates the known binding features of sorafenib and thereby provides a robust framework for evaluating the binding characteristics of novel analogs within the VEGFR-2 active site.

**Fig. 18:**
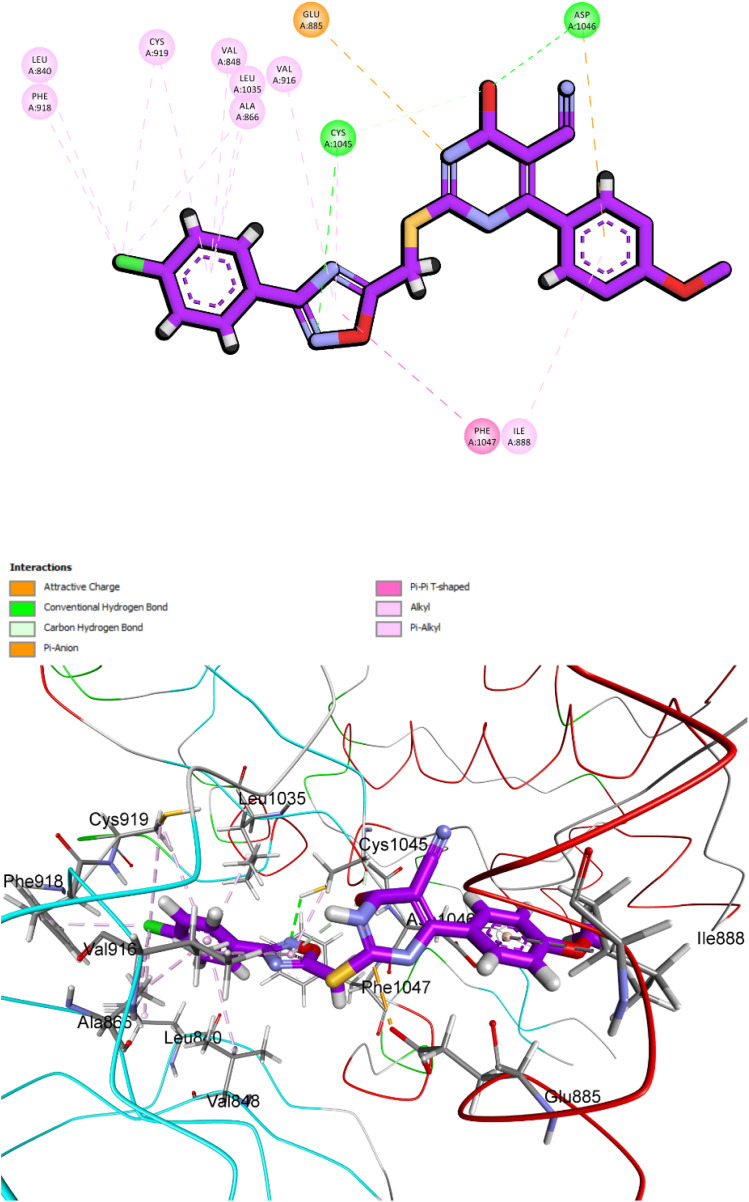
Top: 2D interaction diagram of 10k docked into VEGFR-2 (PDB ID: 3WZE), highlighting hydrogen bonds with Cys1045 and Asp1046, and hydrophobic contacts with Phe918, Val916, and Leu840. Bottom: 3D view of the ligand–receptor complex showing the spatial fit of each pharmacophoric moiety within the VEGFR-2 binding pocket.

Compound 10k, the most potent derivative in the current series, demonstrated exceptional inhibitory activity against VEGFR-2, with an IC_50_ value of 21 nM. This result was corroborated by its favorable docking performance, which yielded a CDOCKER interaction energy score of −7.42 kcal mol^−1^ and an RMSD of 1.61 Å. These values approximate those of the reference inhibitor sorafenib, affirming the strong binding potential of 10k within the VEGFR-2 active site (Fig. 18). Structurally, compound 10k maintains the same four-part pharmacophoric arrangement previously described in its EGFR binding mode: a 1,2,4-oxadiazole ring, a dihydropyrimidine-5-carbonitrile core, a 4-methoxyphenyl group, and a *para*-chlorophenyl moiety. In VEGFR-2, each of these elements plays a distinct and complementary role in stabilizing the ligand within the ATP-binding pocket.

The 1,2,4-oxadiazole ring once again functions as a critical anchoring scaffold. Within VEGFR-2, the oxadiazole engages in conventional hydrogen bonding interactions with Cys1045, one of the key residues lining the hinge region of the kinase. Its planar configuration is preserved by the influence of the methoxy-substituted aromatic ring, allowing the oxadiazole nitrogen to maintain an ideal orientation for interaction. This stabilizing interaction serves as a molecular bridge across the hinge, mimicking the binding mechanism of ATP. The dihydropyrimidine-5-carbonitrile core is deeply embedded in the hydrophilic cavity and contributes significantly to polar stabilization. The carbonyl group and hetero nitrogen atom participate in hydrogen bonding and π–anion interaction, particularly with Asp1046, and Glu885 which are critical for kinase regulation and inhibitor binding. The 4-methoxyphenyl group occupies a lipophilic subpocket and engages in hydrophobic interactions. It interacts notably with Ile888, reinforcing spatial retention. The methoxy substituent not only improves lipophilicity but also promotes favorable electronic distribution across the aromatic system, enhancing the accommodation in the hydrophobic region.

Finally, the *para*-chlorophenyl moiety extends into a separate hydrophobic channel within the VEGFR-2 cleft. This group contributes π–alkyl interactions with residues such as Val848, Leu840, Phe918, Leu1035 and Ala866. The chlorine atom plays a subtle yet important role in modulating both hydrophobic fit and spatial occupation of the lateral binding groove.

Altogether, these four pharmacophoric components synergistically secure compound 10k within the VEGFR-2 ATP-binding site. The interaction map highlights a well-distributed combination of hydrogen bonding, electrostatic, and hydrophobic forces, offering structural validation for the potent biological activity and reinforcing its promise as a dual EGFR/VEGFR-2 inhibitor.

**Fig. 19:**
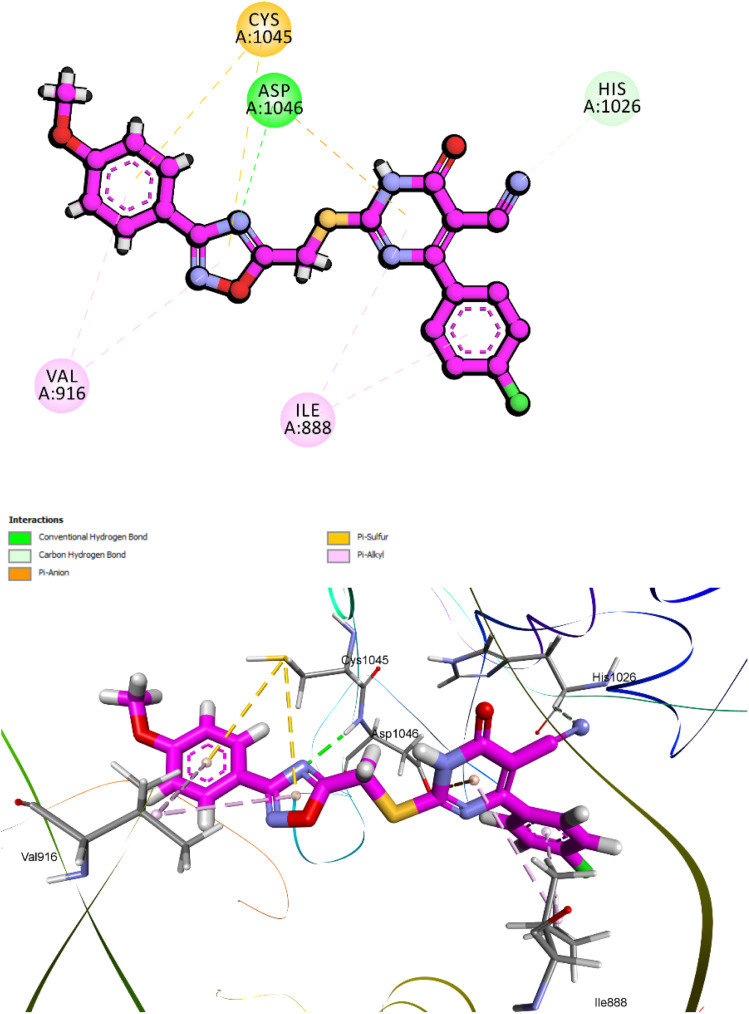
Molecular docking pose of compound 10i within the active site of VEGFR-2 (PDB ID: 3WZE). Compound 10i adopts a stable orientation supported by multiple key interactions: a hydrogen bond and π–anion interaction with, π–alkyl, and π–alkyl interactions.

Compound 10i exhibited a comparable and robust binding mode within VEGFR-2, with a docking energy score of −7.31 kcal mol^−1^ and an RMSD of 1.73 Å. Its interaction network resembled that of 10k but reflected the reversed phenyl substitution, Fig. 19. The 1,2,4-oxadiazole ring scaffold formed a crucial hydrogen bond with Asp1046 and engaged in π–anion stabilization with dihydropyrimidine-5-carbonitrile, anchoring the ligand in the hydrophilic hinge region. The *para*-chlorophenyl moiety maintained hydrophobic π–alkyl contacts with Ile888, while the 4-methoxyphenyl ring engaged in π–alkyl interactions with Val916. Additionally, the methoxy-substituted phenyl group and the 1,2,4-oxadiazole ring scaffold both contributed to a π–sulfur interaction with Cys1045, further enhancing stability within the active site. This synergistic combination of polar, hydrophobic, and sulfur-mediated contacts explains the high docking affinity of 10i in VEGFR-2 and correlates well with its *in vitro* inhibitory performance.

Taken together, these docking studies confirm that both compounds 10k and 10i adopt stable and well-defined binding orientations in EGFR and VEGFR-2, maintaining the hinge-binding hydrogen bond as a central pharmacophoric requirement. The reversal of phenyl substitutions in 10i did not disrupt binding efficiency, as the compound preserved and even complemented critical hydrophobic, electrostatic, and hydrogen-bonding interactions. These results provide compelling structural evidence for the dual inhibitory activity of 10i and reinforce its potential as a promising lead compound alongside 10k in the design of potent EGFR/VEGFR-2 inhibitors.

##### 3.3.1.2. Molecular dynamics (MD) simulations of 10k, 10i and erlotinib with EGFR

Molecular dynamics (MD) simulations were conducted over 100 ns to examine the conformational stability and interaction profiles of compounds 10k, 10i, and the reference drug erlotinib within the ATP-binding site of EGFR using GROMACS 2023.^70^ The initial preparation of protein–ligand complexes was carried out in UCSF Chimera, where all hydrogen atoms were added to maintain proper bond geometries. The CHARMM36 force field was used for the protein, and ligand topologies were generated through CGenFF using the ParamChem web interface.^71,72^ All parameters showed penalty scores <10, confirming high-quality force field compatibility and eliminating the need for manual adjustments. Solvation was performed using a TIP3P water model in a cubic periodic box with a 1.0 nm margin.^73^

To replicate physiological conditions, the system was neutralized and ionized with Na^+^ and Cl^−^ at 150 mM. Following energy minimization using the steepest descent algorithm, the system underwent 100 ps equilibration under NVT and NPT ensembles. Temperature and pressure were maintained at 300 K and 1 bar, respectively, using the V-rescale thermostat and Parrinello–Rahman barostat.^74,75^ A 100 ns production run followed, using a 2 fs timestep, with constraints on hydrogen bonds applied *via* the LINCS algorithm.^76^ Long-range electrostatics were handled by the Particle Mesh Ewald (PME) method with a 10 Å cutoff.^77^

**Fig. 20:**
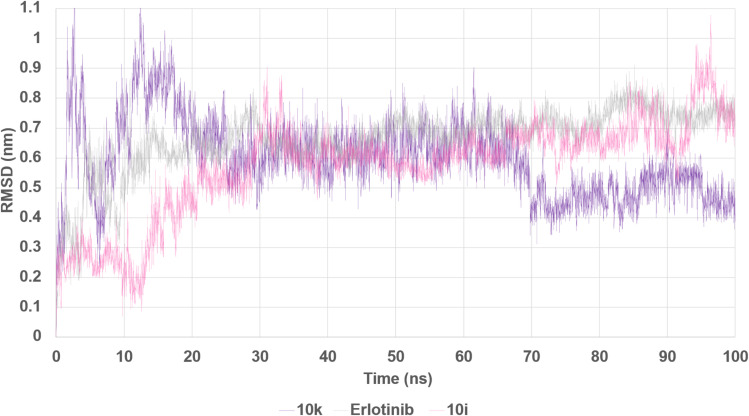
Root Mean Square Deviation (RMSD) of EGFR–ligand complexes over 100 ns MD simulation. The RMSD profiles of compounds 10k (purple), 10i (pink), and erlotinib (gray) in complex with EGFR were monitored to evaluate structural stability over time. Compound 10k exhibited the lowest and most stable RMSD, indicating enhanced conformational rigidity of the complex compared to 10i and erlotinib.

The stability of the complexes was evaluated through several parameters, including root mean square deviation (RMSD), radius of gyration (*R*_g_), root mean square fluctuation (RMSF), hydrogen bond occupancy, and total potential energy. The backbone RMSD analysis (Fig. 20) revealed that all complexes reached equilibrium after an initial equilibration phase within the first 10–20 ns. Notably, the 10k–EGFR complex exhibited the lowest RMSD values throughout the production run, stabilizing within the range of 0.45–0.55 nm and further tightening after 70 ns, indicating a highly stable binding mode. In contrast, compound 10i showed slightly higher RMSD values (0.60–0.70 nm), with sporadic fluctuations observed during the latter part of the simulation, while erlotinib displayed an intermediate RMSD profile with a gradual upward drift in the final third of the trajectory. These findings suggest that compound 10k forms a more rigid and tightly bound complex, consistent with its experimentally observed slightly superior EGFR inhibitory activity relative to 10i.

**Fig. 21:**
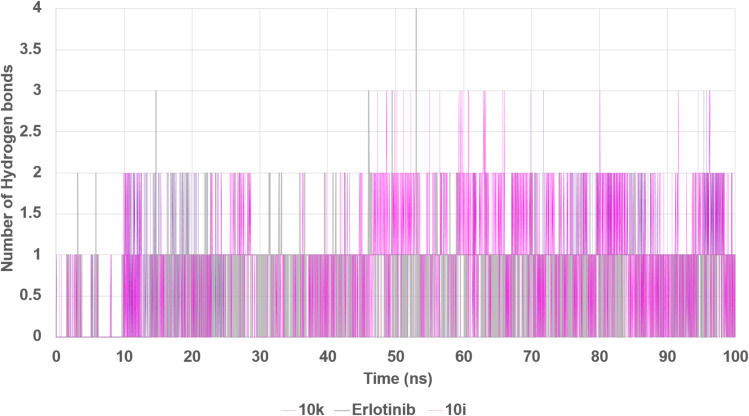
Hydrogen bond analysis between EGFR and bound ligands during 100 ns simulation. The number of hydrogen bonds formed by compounds 10k, 10i, and erlotinib with EGFR was tracked throughout the simulation.

Hydrogen bonding analysis demonstrated that 10i maintained the most frequent polar interactions with the binding site, frequently forming one to two hydrogen bonds, with intermittent periods of up to three concurrent interactions, particularly after 45 ns (Fig. 21). Compound 10k exhibited fewer hydrogen bonds overall but maintained consistent occupancy, suggesting a balance between polar interactions and hydrophobic stabilization, possibly through π–alkyl and π–anion contacts, as supported by docking data. Erlotinib, in comparison, demonstrated a lower hydrogen bond profile, typically maintaining one hydrogen bond throughout the simulation.

**Fig. 22:**
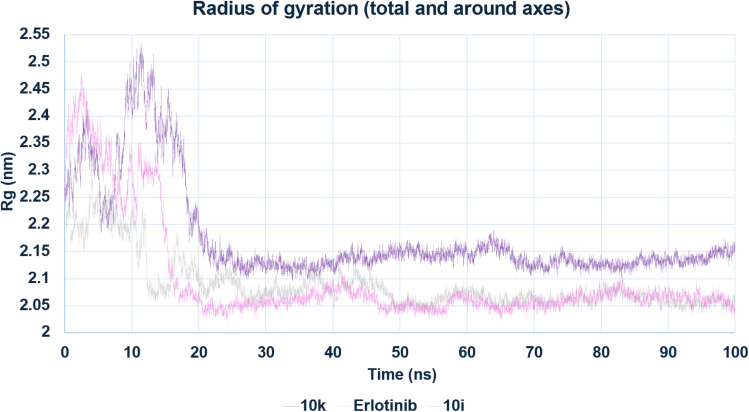
Radius of Gyration (*R*_g_) of EGFR–ligand complexes during MD simulation. The *R*_g_ profiles of the EGFR complexes with 10k, 10i, and erlotinib reflect overall compactness and folding stability.

The radius of gyration (*R*_g_) values for the complexes confirmed their structural compactness and equilibrium stability (Fig. 22). All systems achieved consistent *R*_g_ values by ∼25 ns, with 10i displaying the smallest average *R*_g_ (2.05–2.10 nm), followed by erlotinib (2.08–2.11 nm), and 10k showing slightly higher values (2.12–2.16 nm). The marginal increase in *R*_g_ for 10k is attributed to conformational breathing in the binding site, although this did not translate into decreased overall stability.

**Fig. 23:**
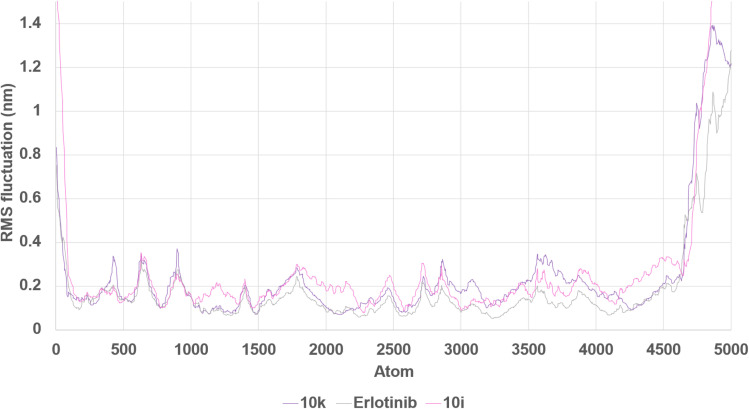
Root Mean Square Fluctuation (RMSF) of Cα atoms in EGFR–ligand complexes. RMSF values per residue reveal local flexibility differences induced by binding of 10k, 10i, and erlotinib. Compound 10k induced the least fluctuation near the active site residues.

Local flexibility, as determined by RMSF (Fig. 23), indicated that compound 10k induced the least atomic fluctuations across the protein, particularly in regions adjacent to the active site. In contrast, 10i induced slightly elevated fluctuations, particularly in loop regions, while erlotinib showed intermediate flexibility. These findings are consistent with the notion that 10k more effectively dampens local motions within EGFR, possibly contributing to longer residence time.

**Fig. 24:**
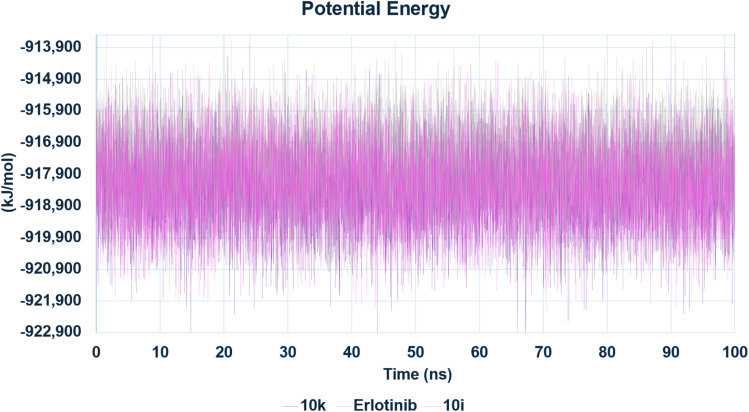
Total potential energy profiles of EGFR–ligand complexes over the simulation time. The energy trajectories of the complexes remained stable with no significant deviations, confirming proper equilibration and thermodynamic stability of the systems. All three ligands—10k, 10i, and erlotinib—exhibited comparable potential energy values throughout the simulation.

The potential energy profiles of all three systems overlapped closely, with no significant drifts or spikes, indicating that all simulations were well equilibrated and energetically stable (Fig. 24).

Taken together, the MD simulations provide compelling evidence that both 10k and 10i are capable of forming stable and persistent interactions with EGFR over extended timescales. However, compound 10k demonstrates superior dynamic characteristics, including lower RMSD and RMSF, which correlate well with its slightly more potent IC_50_ value against EGFR compared to 10i. Erlotinib, although a clinically validated inhibitor, exhibits greater conformational mobility under the same simulation conditions. These findings highlight the favorable structural dynamics of the designed hybrids and support compound 10k as the most stable and promising EGFR inhibitor among the tested compounds.

#### 3.3.2. Quantum mechanical (QM) computations for compound 10k

Quantum mechanical (QM) calculations were employed to complement molecular docking and dynamics simulations by evaluating the electronic structure, charge distribution, and reactivity descriptors of compound 10k, the most potent dual EGFR/VEGFR-2 inhibitor in the current series. These computations provide a deeper understanding of how the electronic features support its observed inhibitory efficacy and receptor binding performance.^78,79^

##### 3.3.2.1. Density functional theory (DFT) analysis of compound 10k

**Fig. 25:**
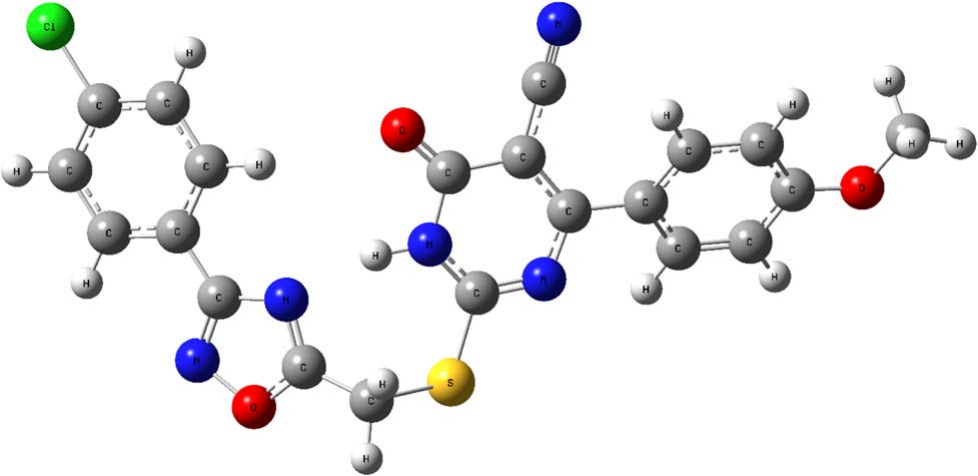
Optimized molecular geometry of compound 10k at the B3LYP/6-311+G(2d,p) level, confirming a minimized energy conformation.

The optimized geometry of compound 10k (Fig. 25) was calculated at the B3LYP/6-311+G(2d,p) level of theory.^80^ Frequency analysis confirmed the absence of imaginary frequencies, validating the structure as a true energy minimum.

**Fig. 26:**
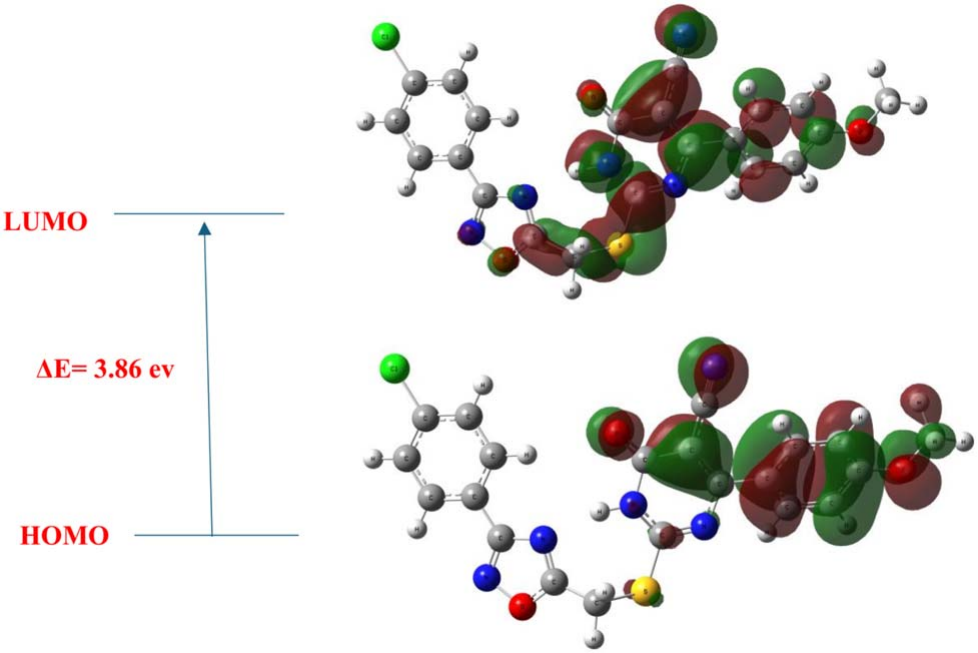
Frontier molecular orbitals of 10k: HOMO (bottom) and LUMO (top), with a Δ*E* of 3.86 eV. HOMO density is concentrated over the pharmacophores (dihydropyrimidine and terminal 4-methoxy phenyl), while LUMO extends toward oxadiazole rings.

The calculated HOMO–LUMO energy gap (Δ*E*) of 3.86 eV (Fig. 26) reflects a moderate reactivity range sufficient to enable receptor interaction without risking premature degradation. The calculated dipole moment of 6.4 debye highlights substantial molecular polarity, which not only promotes aqueous solubility but also enhances directional hydrogen bonding supporting the persistent polar contacts observed throughout the MD simulation. The chemical hardness (*η*) and softness (*σ*) were determined to be 1.93 eV and 0.518 eV^−1^, respectively, reflecting moderate resistance to electronic perturbation and a readiness to engage in polarizable interactions.

These features support adaptive fit of compound 10k into the charged environment of kinase active sites. The HOMO was found to be delocalized over the electron-rich dihydropyrimidine and 4-methoxyphenyl groups, suggesting these regions function as electron donors during interactions consistent with hydrogen bonding and π-donor engagement with critical residues like Met769 (EGFR) and Cys1045 (VEGFR-2). This indicates their potential role as electron-accepting regions during ligand–receptor binding, particularly in π–π stacking, π–anion, and electrostatic interactions—evident in docking studies with residues such as Asp1046, Phe699, and Ile888. Notably, the LUMO extension onto the 4-methoxyphenyl ring reinforces its contribution to hydrophobic stabilization and electronic complementarity with nonpolar subpocket of VEGFR-2.

Collectively, the spatial separation of HOMO and LUMO across distinct pharmacophoric domains mirrors the bifunctional electronic architecture, optimizing it for stable yet adaptable interactions with both EGFR and VEGFR-2.

##### 3.3.2.2. Molecular electrostatic potential (MEP) analysis

**Fig. 27:**
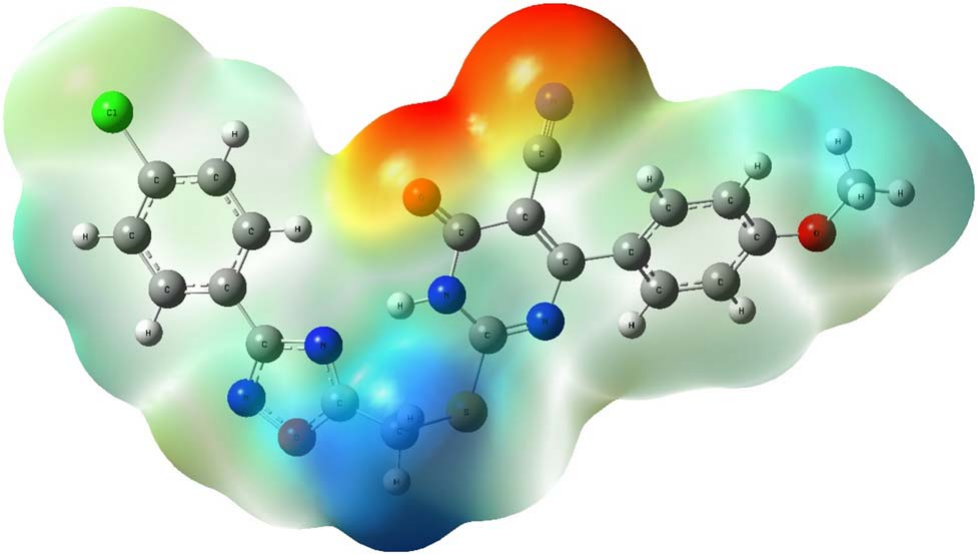
Molecular Electrostatic Potential (MEP) map of compound 10k. Electron-rich regions (red/yellow) align with hydrogen bond acceptor sites, and electron-deficient regions (blue) indicate donor functionality, complementing kinase active-site polarity.

The MEP map of compound 10k (Fig. 27) reveals a clear delineation of electrostatic features relevant to binding. Highly negative potential regions (red) were localized around the nitrile group and carbonyl oxygen atoms, indicating sites of nucleophilic character likely to serve as hydrogen bond acceptors. These findings align with interaction maps from docking, where such moieties formed stable H-bonds with residues like Asp831 (EGFR) and Glu885 (VEGFR-2). In contrast, electron-deficient zones (blue) were detected on the N–H hydrogen atoms of the dihydropyrimidine ring and the aliphatic spacers, suggesting potential hydrogen bond donor roles. Additionally, the *para*-chlorophenyl ring displayed moderately negative electrostatic potential, enhancing its role in halogen bonding and van der Waals stabilization, particularly with Leu764 and hydrophobic residues in both kinases. The 4-methoxyphenyl group, while less polar, contributes electron density to facilitate π–π stacking and hydrophobic embedding, as observed with Phe699 (EGFR) and Ile888 (VEGFR-2).

In summary, the DFT analysis of compound 10k reveals a well-balanced electronic profile characterized by moderate chemical hardness, substantial molecular polarity, and a strategically distributed HOMO–LUMO pattern. These electronic attributes align closely with the experimentally observed binding behavior, supporting its dual inhibitory activity against EGFR and VEGFR-2. The spatial separation of frontier orbitals across key pharmacophoric moieties reinforces the mechanistic insights from docking and MD studies, supporting the rational design of compound 10k as a structurally and electronically optimized scaffold for further preclinical kinase inhibitor development.

### 3.4. ADME studies

A comparative ADME (Absorption, Distribution, Metabolism, and Excretion) evaluation of compounds 10k and erlotinib was conducted using SwissADME to explore their pharmacokinetic viability and drug-likeness in the context of kinase inhibition. Both compounds fully comply with Lipinski's, Ghose's, and Muegge's filters, suggesting a favorable foundation for oral bioavailability. However, 10k marginally violates Veber's and Egan's criteria due to a TPSA of 142.99 Å^2^, exceeding the 140 Å^2^ threshold, which is also observed in erlotinib (TPSA = 142.99 Å^2^). Despite this, both molecules maintain a bioavailability score of 0.55, indicating acceptable oral drug potential.

From a solubility standpoint, 10k and erlotinib are both predicted to be poorly soluble by the SILICOS-IT model and moderately soluble by ESOL and Ali models, reflecting the need for formulation support to enhance solubility. Notably, 10k shows low GI absorption, in contrast to erlotinib which exhibits high GI absorption, likely due to the higher polarity and larger molecular size of 10k. Additionally, 10k is capable of permeating the blood–brain barrier (BBB), whereas erlotinib is not, indicating potential for CNS activity or associated side effects. Regarding cytochrome P450 interactions, 10k shows inhibition of CYP2C9 and CYP3A4, whereas erlotinib exhibits a broader inhibitory profile, affecting CYP1A2, CYP2C19, CYP2C9, CYP2D6, and CYP3A4. This suggests that 10k may offer a more selective metabolic profile with fewer drug–drug interaction risks, which may be relevant in guiding further preclinical development and optimization in clinical settings. In terms of lipophilicity, the consensus Log *P*_o/*x*_ for 10k is 3.66, slightly higher than that of erlotinib (3.20), suggesting greater membrane affinity but potentially lower solubility. However, Log *K*_p_ (skin permeability) values for both are comparable (−6.37 cm s^−1^ for 10k*vs.* −6.35 cm s^−1^ for erlotinib), implying similar topical absorption limitations.

Finally, 10k shows no PAINS alerts, a moderate synthetic accessibility score (3.36), and no predicted P-gp substrate behavior, differentiating it from erlotinib, which may be influenced by efflux mechanisms. Overall, these ADME findings reinforce the pharmacokinetic promise of compound 10k, particularly its metabolic selectivity and acceptable oral drug profile, suggesting its potential as a preclinical lead compound with a differentiated pharmacokinetic profile relative to erlotinib. However, further *in vivo* and pharmacodynamic studies are needed to confirm these predictions.

### 3.5. Structure activity relationship (SAR) of compounds 10a–l

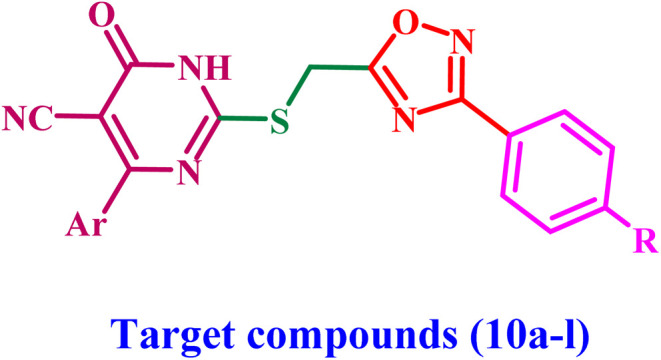



#### 3.5.1. EGFR inhibitory activity

1. The 1,2,4-oxadiazole ring acts as the core anchoring motif, forming an important hydrogen bond with the hinge residue Met769, a conserved interaction required for successful ATP-competitive kinase inhibition. This ring is kept in a planar configuration, which is essential for good alignment within the tight hinge region.

2. In addition, the 1,2,4-oxadiazole ring forms a π-π stack with Ala719 and Leu820.

3. The dihydropyrimidine-5-carbonitrile core takes up the center polar portion of the EGFR binding pocket. This scaffold aids in molecule recognition by hydrogen bonding and electrostatic interactions, particularly through its cyano group, which interacts favorably with residue Asp831, improving binding stability and directional specificity.

4. Additionally, The ring is pointed toward hydrophobic residues like Leu694, which participate in π-alkyl interaction.

5. The methoxy group on the phenyl ring of the dihydropyrimidine moiety is essential for activity. The methoxy group reinforces the 1,2,4-oxadiazole moiety planarity within the EGFR pocket site, stabilizing the oxadiazole orientation *via* electronic and steric effects. The methoxy substituent supports planar geometry while also contributing to hydrophobic and π-π stacking interactions with Phe699 and forming π-alkyl contacts with Val702.

6. The *para*-chlorophenyl moiety of the 1,2,4-oxadiazole motif is also required for action. The chlorophenyl moiety extends into a hydrophobic pocket at the gatekeeper region. The chlorine atom promotes van der Waals and halogen bonding interactions with residues such as Leu764 and Glu738, thereby filling the lateral volume of the binding site and further anchoring the ligand *via* nonpolar stabilization.

#### 3.5.2. VEGFR-2 inhibitory activity

1. The 1,2,4-oxadiazole ring once again functions as a critical anchoring scaffold. Within VEGFR-2, the oxadiazole engages in conventional hydrogen bonding interactions with Cys1045, one of the key residues lining the hinge region of the kinase.

2. The dihydropyrimidine-5-carbonitrile core is deeply embedded in the hydrophilic cavity and contributes significantly to polar stabilization. The carbonyl group and hetero nitrogen atom participate in hydrogen bonding and π–anion interaction, particularly with Asp1046, and Glu885 which are critical for kinase regulation and inhibitor binding.

3. The 4-methoxyphenyl group occupies a lipophilic subpocket and engages in hydrophobic interactions. It interacts notably with Ile888, reinforcing spatial retention. The methoxy substituent not only improves lipophilicity but also promotes favorable electronic distribution across the aromatic system, enhancing the accommodation in the hydrophobic region.

4. Finally, the *para*-chlorophenyl moiety extends into a separate hydrophobic channel within the VEGFR-2 cleft. This group contributes π–alkyl interactions with residues such as Val848, Leu840, Phe918, Leu1035 and Ala866. The chlorine atom plays a subtle yet important role in modulating both hydrophobic fit and spatial occupation of the lateral binding groove.

## 4 Conclusion

In conclusion, a novel series of dihydropyrimidine-5-carbonitrile/1,2,4-oxadiazole hybrids (10a–l) was synthesized as dual inhibitors of EGFR and VEGFR-2, exhibiting apoptotic antiproliferative and antioxidant properties. Compounds 10k and 10l were identified as the most effective antiproliferative compounds potentially acting as dual inhibitors of EGFR and VEGFR-2. Computational analyses confirmed 10k as the most stable, selective, and well-oriented dual EGFR/VEGFR-2 inhibitor, with 10i also showing favorable profiles. Docking and MD simulations validated superior binding orientation and dynamic stability of 10k within the kinase active sites. Additionally, DFT and MEP analyses underscored its electronic reactivity, and ADME predictions supported its drug-likeness and metabolic suitability. These integrated results strongly position 10k as the leading candidate for further development in kinase-targeted cancer therapy. While the current study provides comprehensive *in vitro* and *in silico* evaluation of compound 10k, its biological activity, pharmacokinetic behavior, and safety profile remain to be confirmed through *in vivo* studies. As such, these results should be interpreted as preliminary and exploratory, pending further validation in animal models.

## Conflicts of interest

The author disclosed there were no potential conflicts of interest.

## Supplementary Material

RA-015-D5RA05685C-s001

## Data Availability

The authors declare that the data supporting the findings of this study are available within the supplementary information (SI) materials. Supplementary information is available. See DOI: https://doi.org/10.1039/d5ra05685c.
